# The Same against Many: AtCML8, a Ca^2+^ Sensor Acting as a Positive Regulator of Defense Responses against Several Plant Pathogens

**DOI:** 10.3390/ijms221910469

**Published:** 2021-09-28

**Authors:** Xiaoyang Zhu, Julie Mazard, Eugénie Robe, Sarah Pignoly, Marielle Aguilar, Hélène San Clemente, Emmanuelle Lauber, Richard Berthomé, Jean-Philippe Galaud

**Affiliations:** 1 College of Horticulture, South China Agricultural University, Guangzhou 510642, China; 2 Laboratoire de Recherche en Sciences Végétales, Université de Toulouse, CNRS, UPS, Toulouse INP, 24, Chemin de Borde Rouge, 31320 Auzeville-Tolosane, France; xiaoyang_zhu@scau.edu.cn (X.Z.); julie.mazard@lrsv.ups-tlse.fr (J.M.); robe@lrsv.ups-tlse.fr (E.R.);sarah.pignoly@gmail.com (S.P.); marielle.aguilar@lrsv.ups-tlse.fr (M.A.); sancle@lrsv.ups-tlse.fr (H.S.C.); 3 Laboratoire des Interactions Plantes-Microorganismes-Environnement, Université de Toulouse, INRAE, CNRS, 31326 Castanet-Tolosan, France; emmanuelle.lauber@inrae.fr

**Keywords:** *Arabidopsis thaliana*, calcium signaling, calmodulin-like protein, multi-pathogens, plant immunity, *Ralstonia solanacearum*, *Phytophtora capsica*, *Xanthomonas campestris*

## Abstract

Calcium signals are crucial for the activation and coordination of signaling cascades leading to the establishment of plant defense mechanisms. Here, we studied the contribution of CML8, an Arabidopsis calmodulin-like protein in response to *Ralstonia solanacearum* and to pathogens with different lifestyles, such as *Xanthomonas campestris* pv. *campestris* and *Phytophtora capsici*. We used pathogenic infection assays, gene expression, RNA-seq approaches, and comparative analysis of public data on *CML8* knockdown and overexpressing Arabidopsis lines to demonstrate that CML8 contributes to defense mechanisms against pathogenic bacteria and oomycetes. *CML8* gene expression is finely regulated at the root level and manipulated during infection with *Ralstonia*, and *CML8* overexpression confers better plant tolerance. To understand the processes controlled by CML8, genes differentially expressed at the root level in the first hours of infection have been identified. Overexpression of *CML8* also confers better tolerance against *Xanthomonas* and *Phytophtora,* and most of the genes differentially expressed in response to *Ralstonia* are differentially expressed in these different pathosystems. Collectively, CML8 acts as a positive regulator against *Ralstonia solanaceraum* and against other vascular or root pathogens, suggesting that CML8 is a multifunctional protein that regulates common downstream processes involved in the defense response of plants to several pathogens.

## 1. Introduction

In their environment, plants have to face many constraints in order to carry out their development and reproduction cycles. These stressful conditions induce on the plant varied responses depending on the biotic and abiotic stimuli detected [1,2]. Crop losses worldwide due to these stresses are estimated at hundreds of billions of dollars every year [3]. Moreover, in the context of global changes, climatic scenarios predict their increase due to more frequent and severe epidemies [4,5].

Plant defense mechanisms against a limited number of pathogens have been extensively studied. They depend on physical responses that involve specific structures, such as the plant cell wall or the presence of the cuticle, and two layers of immune responses. The first layer involves pathogen-associated molecular patterns (PAMP) recognition by plant cell surface receptors, initiating a signaling cascade leading to the PAMP-triggered immunity (PTI). PTI confers a basal resistance level to a broad spectrum of pathogens. To overcome PTI, some pathogens produce effectors that interfere with host defense responses. Such effectors can be recognized by intracellular resistance proteins which activate the second layer of plant defense, called the effector-triggered immunity (ETI) [6], which often restricts further spread of the pathogen through a localized programmed cell death [7]. Rather than being composed of distinct layers of defenses, plant resistance has been suggested to be a continuum between PTI and ETI, with each layer sharing components and having been demonstrated to influence each other [8,9]. The more complex nature of plant immunity is illustrated by the diversity of defense response phenotype observed in natural and field conditions [10,11] and the recent study of the genetic basis of quantitative disease resistance (QDR) suggests that this phenomenon is explained by a polygenic architecture [12,13].

The increase of calcium (Ca^2+^) concentration in the cytosol is one of the earliest responses induced upon perception of a pathogen by plants [14,15]. It has been proposed to be responsible for the coordination and activation of signaling cascades leading to the establishment of appropriate cellular responses [16,17]. Indeed, the application of lanthanum, an inhibitor of Ca^2+^ influx, suppresses plant response linked to the resistance gene *RPM1* during an infection with *Pseudomonas syringae* pv. *tomato* (*Pst*) [18]. If complex spatiotemporal patterns of Ca^2+^ influx (frequency, amplitude, duration) within the cell are thought to encode information related to the initial stimuli, to become interpretable information, these Ca^2+^ variations need to be decoded by Ca^2+^-binding proteins to produce the appropriate responses. Interestingly, plants have a large repertoire of specific Ca^2+^ sensors such as the calmodulin (CaM), Ca^2+^-dependent protein kinases (CDPKs), the calcineurin-B-like (CBLs) and the calmodulin-like (CMLs) proteins, indicating that plants possess specific Ca^2+^ signaling components [19,20,21].

While CMLs are related to the typical CaM, their physiological roles remain mostly unknown [17]. In Arabidopsis, there are 50 CMLs that all contain Ca^2+^ EF-hand binding motifs but no other known functional domain. Interestingly, genetic approaches showed that CMLs are involved in abiotic and/or biotic stress responses. For example, the vacuolar AtCML18 is able to interact with the Na^+^/H^+^ antiporter AtNHX1 to regulate plant response to salt stress [22]. The plasmodesmal-localized CML41 promotes callose deposition at the plasmodesmata level following flagellin perception, showing that CML41 acts as a positive regulator of defense against *Pst* [23]. The Arabidopsis CML9 has a dual role and acts either as a negative regulator of responses linked to ABA and drought [24] or as a positive regulator of responses to *Pst* through the PTI flg22-dependent pathway [25]. All these data indicate that CMLs do not have total functional redundancy and could act as part of signaling crosstalk to coordinate plant responses to multiple environmental stresses by being positive and/or negative regulators [24].

More recently, it was shown that CML8, closely related to CML9, is also a positive regulator of plant immunity against the leaf mesophyll pathogen *Pst* [26]. Unlike to *CML9*, *CML8* gene is preferentially transcribed at the root level. This raises the question of its putative involvement in plant responses to other pathogens and particularly for soilborne pathogens. To answer this question, the contribution of CML8 to plant defense was analyzed following infection by the bacterium *Ralstonia solanacearum* (*Rs*)*. Rs* is a major phytopathogenic bacterium, present in the soil, that attacks more than 200 plant species in tropical, subtropical, and warm temperate areas worldwide [27]. *Rs* infects the plant at the emergence site of the lateral roots (LRs) and root tips [28], then invades the xylem vessels to spread towards the aerial parts of the plant through the vascular system and cause plant wilting [29,30].

In this work, we show that *CML8* expression is tightly regulated at the root level and manipulated by *Rs* during the early steps of infection. Using *CML8* overexpressing and knockdown transgenic lines, we show that overexpression of *CML8* confers a resistance to this bacterium. To elucidate the molecular mechanisms controlled by CML8 in the early stages of plant response to *Rs,* genes differentially expressed at the root level within the first hours of infection were identified, using an RNA-sequencing (RNA-seq) strategy. Interestingly, the inoculation of *CML8* transgenic lines with other pathogenic microorganisms with different lifestyles and the study of the expression profiles of our top list of differentially expressed genes (DEGs) strongly suggest CML8 and downstream actors as core elements of the plant defense response to several pathogens.

## 2. Results

### 2.1. CML8 Gene Expression Is Finely Tuned during LR Development and Exogenous Hormones Application

**Figure 1:**
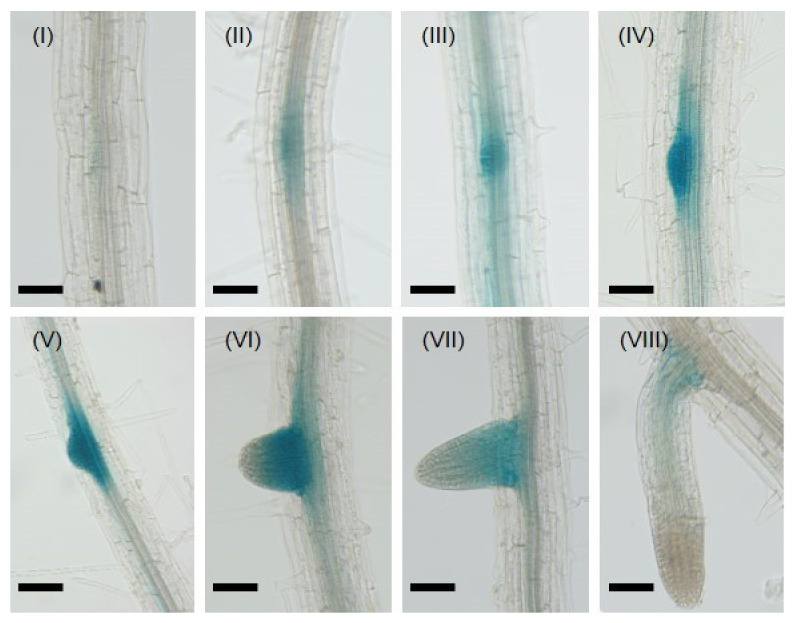
*AtCML8* is transiently expressed during lateral root formation. GUS activity staining was observed on seven days-old *promoter CML8::uidA* transgenic Arabidopsis seedlings. (**I**) to (**VIII**) photos correspond to the eight developmental stages as described in [31]. Bars: 50 µm.

The expression of *CML8* throughout the whole plant had previously been explored by Zhu et al. [26] and showed that *CML8* highest level of expression was found in the LRs, compared with other plant tissues. To investigate *CML8* root-expression pattern more deeply, we focused on the eight developmental stages of LR formation as defined in Arabidopsis by Casimiro et al. [31], using *promoter CML8::GUS* reporter transgenic lines (Figure 1).

*CML8* expression was first observed at the first stage of the LR formation process when two pericycle founder cells located next to the xylem started to divide. Then, gradually increasing GUS staining was visible when the cells forming the LR primordium actively divided asymmetrically and expanded radially towards the endodermis. GUS staining was most intense during stages IV and V of LR formation when the LR primordium had broken through first the endodermis, then cortical cells, but not yet through the epidermal layer. As soon as the LR emerged from the epidermis, *CML8* expression stayed confined to the LR emergence while the LR continued to grow. *CML8* expression was not detected in primary roots and in root tips, showing that it is limited to the early stages of LR formation. LR emergence is a process that greatly impacts the primary root structure and integrity and leaves gaps around its sites that become entry points for pathogens inside the plant [32]. As LR initiation is controlled by auxin, *CML8* gene expression was analyzed following exogenous auxin application (IAA, 1 µM) using *promoter CML8::GUS* reporter transgenic lines (Supplementary Materials, Figure S1) after 3 h, 6 h, and 24 h. In the conditions tested, no evidence indicated that *CML8* gene expression was induced or repressed by IAA. We next analyzed *CML8* expression in response to other hormones, including 1-Aminocyclopropanecarboxylic acid (ACC; ethylene precursor), brassinosteroids (BR), salicylic acid (SA), gibberellic acid (GA), methyl jasmonate (MJ), and GR24 (strigolactone analog), in similar conditions to those used for auxin (Supplementary Materials, Figure S1). As with IAA, ABA and MJ did not modify *CML8* expression pattern and level, whereas ACC, BR, SA, and GA treatments slightly stimulated *CML8* expression in a rapid but transient manner. GR24, a strigolactone analog, seemed to activate *CML8* expression after 24 h. Collectively, these results showed that *CML8* gene expression is finely tuned during LR development and hormonal control in this process cannot be excluded.

### 2.2. CML8 Gene Expression Increases in Roots Following Rs Inoculation

**Figure 2:**
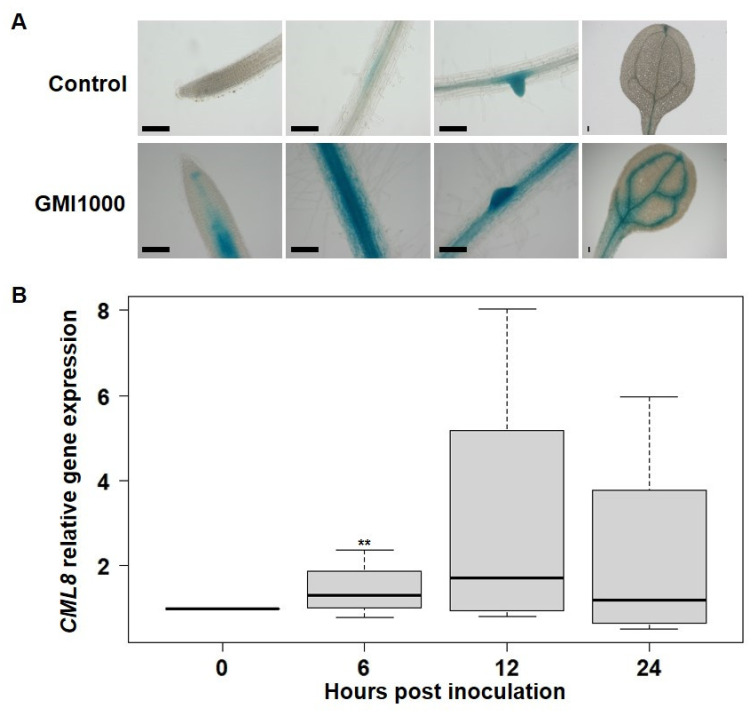
*CML8* gene expression is induced in response to *Rs* inoculation. (**A**) Expression pattern of *CML8* using eight days-old *promoter CML8::uidA* transgenic Arabidopsis plants. GUS staining was performed 6 h after 1·10^7^ cfu·mL^-1^ *Rs* GMI1000 strain or mock treatment (Control) inoculation. Bars: 100 μm. (**B**) RT-qPCR analysis of *CML8* transcript levels in roots of Arabidopsis plants following inoculation with *Rs* GMI1000 strain. The expression level of *CML8* corresponds to the relative expression level compared to the *EF1-ɑ* reference gene. This experiment was performed four times on three independent replicates. Statistical analyses were performed using Student’s t-test and significant difference was found with *p*-values < 0.05 (** < 0.01).

As CML8 was shown to act as a positive regulator of plant defense responses against *Pst* [26], and considering that *CML8* expression was restricted to LR initiations that are considered an entry point for many plant soil pathogens, we investigated the contribution of CML8 in response to the soilborne bacterium *Rs* [33]. Firstly, we analyzed the expression levels of *CML8* 6 h after inoculation with *Rs* GMI1000 WT strain using the *promoter CML8::GUS* reporter lines. As shown in Figure 2A, *Rs* inoculation strongly induces the expression of *CML8* mainly in the vascular tissues of roots and leaves that correspond to the multiplication areas of this vascular bacterium. We confirmed this data using RT-qPCR and showed that the expression of *CML8* is on average respectively 1.4-fold, 3-fold and 2.2-fold higher at 6 hpi, 12 hpi and 24 hpi following bacteria inoculation compared to control conditions. Increase of *CML8* expression is transient and decrease within 24 hpi (Figure 2B). Collectively, these results question a putative involvement of CML8 in plant responses during the early stages of *Rs* infection.

### 2.3. CML8 Acts as a Positive Regulator of the Plant Defense Responses Following Rs Inoculation

**Figure 3:**
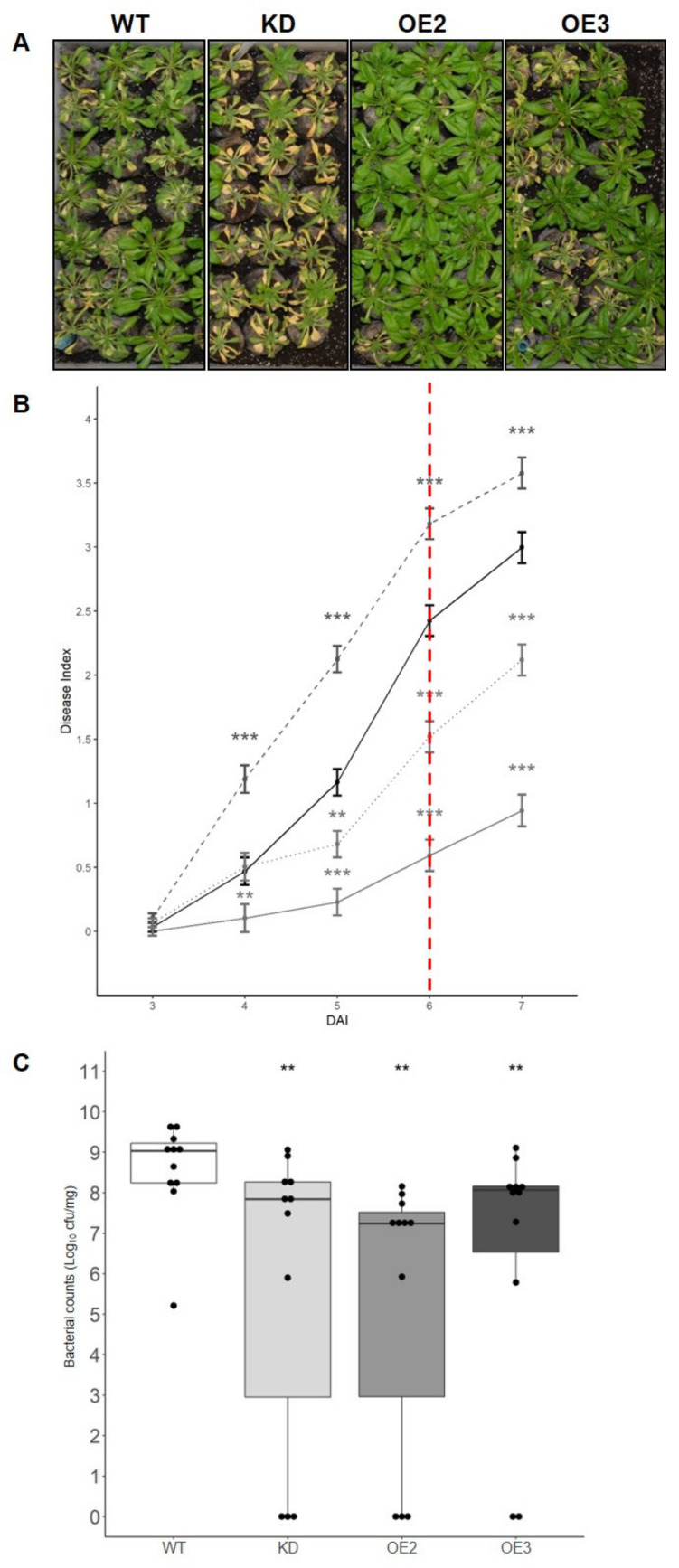
Comparative analysis of the susceptibility of *CML8* genotypes upon inoculation with *Rs* GRS100 strain that is deleted for the effector PopP2 complemented with PopP2. Cut roots of four weeks-old plants of WT, *CML8* KD line and OE lines were inoculated with a suspension of 1·10^7^ cfu·mL^-1^ of *Rs* GRS100 strain. (**A**) Photographs of disease symptoms were taken at nine dai. (**B**) Disease symptoms index are shown from three dai to seven dai. The solid black line, the dashed dark grey line, the solid grey line and the dashed light grey line represent WT, *CML8* KD, OE2 and OE3 lines, respectively. The red dotted line indicates the day when bacteria *in planta* quantification was done. Error bars = SEM were obtained from 69 inoculated plants in three independent biological replicates. (**C**) Quantification of *in planta* bacterial growth performed at six dai of *Rs* GRS100 strain complemented with PopP2 inoculation. Statistical analyses were performed as described in Materials and Methods section and significant difference was found with *p*-values < 0.05 (*** < 0.001, ** < 0.01).

To investigate the role of CML8 in the plant responses to *Rs*, we inoculated plants previously characterized for the down- (KD) and upregulation (OE2 and OE3) of *CML8* expression as well as the WT susceptible control [26]. Initially, to determine whether the *RRS1* gene could play a role in the CML8-related response to *Rs*, phenotyping was carried out with different strains of *Rs*, mutated in PopP2 or complemented with different forms of the PopP2 effector. No such involvement was uncovered, indicating that CML8-mediated responses lean more towards basal defense and PTI than ETI. It is the reason why the results presented in Figure 3 correspond to plants inoculated with the GRS100 *Rs* mutant strain, mutant harboring a mutation in PopP2, complemented with the native form of PopP2 and that behaves similarly to the WT strain GMI1000 (Supplementary Materials, Figure S2). The results, representative of three independent biological replicates, showed, even if the *CML8* KD was not always significantly different from WT, that wilting symptoms appeared faster, indicating it was more susceptible than the WT. By contrast, the OE transgenic lines always displayed less symptoms than WT in response to *Rs* inoculation during the whole experiment, these differences being significant (Figure 3A,B).

To elucidate if these discrepancies could be correlated to a defense response, the bacterial multiplication in planta was quantified (Figure 3C) on leaves at 6 dai when the difference in disease index was the greatest compared to the WT. Interestingly, bacterial growth was significantly decreased in all transgenic lines with the lowest multiplication observed in the OE2 line. Altogether, these data supported that the overexpression of *CML8* contributed to the plant resistance to *Rs*.

### 2.4. Rs Induces a Rapid and Progressive CML8-Dependent Transcriptional Reprogramming

To decipher the early molecular events depending on CML8 that control the plant responses to *Rs*, the root transcriptome of KD, OE2, and WT plants inoculated or not with the bacterium at 6, 12, and 24 hpi was analyzed using an RNA-seq approach. A list of DEGs was established based on an FDR correction at 5% (Supplementary Materials, Table S1).

**Figure 4:**
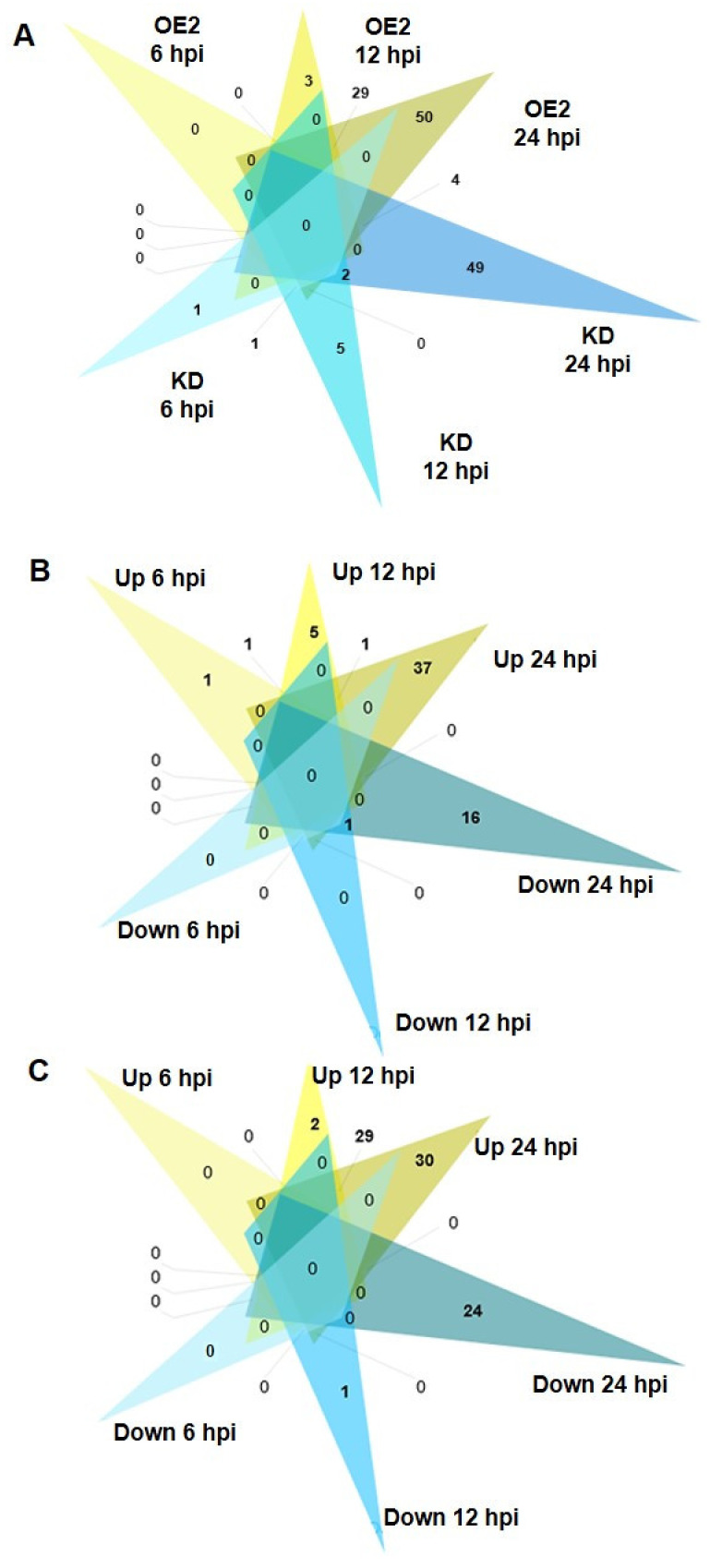
Number of differentially expressed genes in roots found specifically during an infection with *Rs* GMI1000 strain based on a 5% FDR. (**A**) Numbers in Venn diagrams indicate the number of DEGs found at each time point in the roots of *CML8* KD (blue color) and OE2 plants (yellow color) compared to WT plants. (**B**) Number of up- (yellow) and down-regulated DEGs (blue) in roots specific to each time point of the kinetic or overlapping between time point in KD line. (**C**) Number of up- (yellow) and down-regulated DEGs (blue) in roots specific to each time point of the kinetic or overlapping between time point in OE2 line.

Overall, with FDR at 5%, 151 DEGs were identified when compared to WT, with 63 and 92 genes in KD and OE2 lines, respectively (Supplementary Materials, Table S1, Figure 4A). The 3, 9, 56 DEGS and 6, 38, 89 were identified at 6, 12, and 24 hpi in *CML8* KD and OE2 lines, respectively (Supplementary Materials, Table S1) with up- or downregulated DEGs at each kinetic point presented in Figure 4B,C, respectively. Little overlap is observed between the DEGs lists identified in KD and OE2 lines (Figure 4A). Moreover, once induced or repressed at the first kinetic time point, a progressive increase of the down- or upregulation of these genes is observed, while being accompanied by the identification of new DEGs, leading to a greater number of DEGs at 24 hpi in KD (Figure 4B) as well as in OE2 (Figure 4C). To verify up- and downregulated DEGs, seven genes either induced or repressed at different kinetic time points were used for RT-qPCR experiments using specific primers (Supplementary Materials, Table S5). These genes were chosen according to their expression profiles and correspond to candidate genes detailed hereinafter. The RT-qPCR data (Supplementary Materials, Figure S3) were consistent with RNA-seq analysis.

### 2.5. Molecular Pathways Activated or Repressed during Rs Infection in CML8 Genotypes

**Figure 5:**
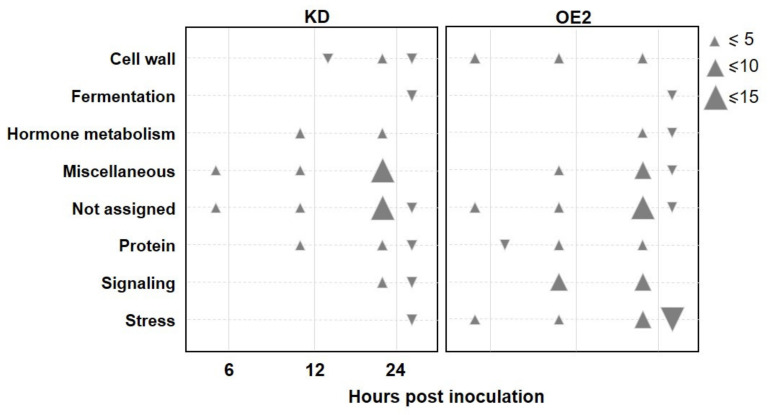
Functional categories significantly over-represented for CML8-dependent DEGs (Table S1) following *Rs* infection in *CML8* KD and OE2 lines, obtained with MapMan Classification Super Viewer. The down- and up arrows correspond to down-regulated DEGs and up-regulated DEGs, respectively. The size of triangle indicates the number of DEGs in each category.

We used the MAPMAN classification function to associate a function to the DEGs from the two lists and analyzed the co-expression or interaction networks in which they could be associated. With a 5% FDR correction, eight functional categories significantly overrepresented were identified in both KD and OE2 lines, corresponding to genes involved in cell wall, fermentation, miscellaneous, protein synthesis or degradation processes, hormone metabolism, stress response, and signaling (Figure 5).

Notably, while the genes assigned to these functional categories were not the same, or their expression was differentially regulated between transgenic lines, a greater number of genes involved in signaling and stress response processes were regulated in the *CML8* OE2 (Figure 5, Supplementary Materials, Table S1). Consistently, many induced genes are involved in defense response to pathogens, and in particular to *Rs*, such as *PAD4*, *EDS1,* and *EDS5*, as well as several genes coding for characterized NLRs, such as SNC1 and RPP1. In addition, several genes also code for factors known or predicted to be involved in Ca^2+^ signaling, such as CML41, CDPK22, and CRK17. To assist in the selection of the most relevant candidate DEGs, we used the GeneMANIA Cytoskape app to construct the gene–gene functional interaction network from our list and identify the most-related genes (Supplementary Materials, Figure S4).

Based on the relevance of the functional category, the predicted function, and co-expression network, seven promising candidate genes were selected for further analyses. They corresponded to cell wall-related genes strongly induced in OE2 line and previously demonstrated to participate in host defense against pathogens: the plant natriuretic peptide A (PNP-A), also named EGC2 (*At2g18660),* upregulated at 12 and 24 hpi, and the lipid transfer protein *LTP4.4* gene (*At5g55450),* induced at 6, 12, and 24 hpi [34,35]. These two genes were co-expressed with three other DEGs, the receptor-like protein *RLP41* (*At3g25010*), strongly upregulated in OE2 line, the nucleoside diphosphate protein *NUDT6* (*At2g04450*) and XB3 ortholog 4 in *Arabidopsis thaliana XBAT34* (*At4g14365*), also highly induced in *CML8* OE2 plants at 12 and 24 hpi. NUDT6 is important for SA-mediated immune responses through several resistance genes such as *NPR1* and *EDS1* [36]. XBAT34 was highly similar to the SAR key regulators NPR1 or NIM1 and whose expression is repressed by BAK1 [37]. As primary metabolism such as ethanol fermentation is often described to be altered during infection by soil pathogens [38], but also because this is one of the genes strongly repressed at 24 hpi in OE2 line, we selected *At4g33070* encoding the pyruvate decarboxylase PDC1. Finally, the *JAL23* (*At2g39330*) gene was retained because it encodes a protein belonging to the Jacalin lectin family, known to participate in the perception of many environmental signals [39], and is one of the most highly induced genes in KD line at 24 hpi.

### 2.6. Comparison with Publicly Available Transcriptomic Data

To determine if the list of 151 DEGs was specific to the plant response to *Rs* or had been identified more broadly in other experiments, it was compared (Supplementary Materials, Table S1) to publicly available transcriptome studies on abiotic and biotic perturbations and elicitor treatments performed either using microarray or RNA-seq approaches (Supplementary Materials, Figure S5). Using hierarchical clustering, similar expression patterns were found between the CML8-controlled genes and a majority of DEGs from the biotic subselection. For instance, the major part of the upregulated DEGs was also induced in studies where WT and mutant of genes involved in secondary metabolism or coding for signaling proteins and PTI actors (*rpp4*, *eds1*, *pad4*, *sid2*) are infected with various phytopathogens such as the necrotrophic fungi *Sclerotinia sclerotiorum* and *Plectosphaerella cucumerina*, the biotrophic oomycete *Hyaloperonospora arabidopsidis,* and the bacterium *Pst*. Interestingly, a strong similarity was found between our genes list and a study looking at the roots transcriptomic response of Arabidopsis to the oomycete *Phytophtora parasitica* in the early stages of the infection [40]. Such an overlap between the biotic stress responses supported that our list of genes was part of the systematic host defense response to an infection with different pathogens and that CML8 may act as a hub to integrate environmental signal related to multiple living organisms. Furthermore, a strong correlation found with elicitor of plant defenses studies predominantly concerned PAMPs such as flg22 [41,42], elf18 [43], chitin [41], or oligogalacturonides known to be released upon *B. cinerea* infection [44], and LRR receptor kinases such as PEPR1/PEPR2 or BAK1 [43] involved in PAMPs perception. Together, this suggested that CML8-dependent plant response leant more towards basal immunity. However, correspondence was less important with abiotic stress studies and no overall tendency was detected towards the developmental studies.

We then compared the DEGs lists with genes that participate in Ca^2+^ perception and signaling and to PTI and ETI regulation (Supplementary Tables S2–S4). Comparisons showed that numerous genes were shared and significantly enriched in Ca^2+^ actors and genes regulated by Ca^2+^ signals [45], such as calreticulin, CMLs, CBLs, CDPKs, and CIPKs. Significant enrichment was also found with studies looking at PTI responses triggered by the hemibiotrophic oomycete *Phytophtora parasitica* [46], the biotrophic fungus *Golovonomyces orontii* [47], the hemibiotrophic fungus *Fusarium oxysporum* [48], and the bacterium *Pst* [49,50]. The highest percentages of overlap between studies (Supplementary Materials, Table S4) were observed with flg22, elf18, and oligogalacturonides PAMP treatments [43,51] that activate typical PTI responses such as signaling cascades involving CDKs, RLKs, WAKs, and MAPKs, SA signaling and camalexin accumulation, expression of marker genes such as *EDS1*, *PAD4*, and *PR1*, and cell wall modifications. Many signaling pathways are shared between PTI and ETI; one of these is the EDS1 signaling pathway [52]. Consistently, we found a significant overlap between our list and studies looking at ETI responses such as defenses elicited by *Pst* avirulent genes *avrRpm1*, *avrRps4*, *avrRpt2,* and *avrPphB* [53,54], SA-independent EDS1 signaling was implicated in resistance to biotrophic and hemibiotrophic pathogens [53] and the necrotrophic bacterium *Erwinia amylovora* [52].

A significant overlap was obtained between our DEGs lists and studies focused on *Rs* infections. Strikingly, while comparing with transcriptome data of leaves of four-week-old Arabidopsis root, inoculated at 6, 12, and 24 hpi and 5 and 8 dai [55], genes involved in basal resistance were found enriched at five dai but not at 12 and 24 hpi. This result could be explained by the fact that Hu et al. [55] used leaves to perform their analysis, while we used roots. We also found significant overlap with studies carried out on Arabidopsis secondary cell wall mutants that displayed enhanced resistance to *Rs*: the *wat1* mutant, in which the resistance is regulated by SA and indole metabolism and efficient against multiple pathogens including *Xcc* [56] *and irx5–5* and *irx1–6* secondary cell wall mutants [57]. We noted that 27% and 35% of KD and OE2 DEGs, respectively, were the same as DEGs identified in roots of seven-day-old seedlings 96 h after being in vitro infected by *Rs* [58]. A significant overlap was also observed in Arabidopsis WT and *rrs1–1* mutants at 2, 4, 6, and 8 h after the leaves were infiltrated by *Pseudomonas* delivering, through its type III secretion system, the PopP2 effector, known to induce ETI when perceived by the immune receptor pair RSP4/RRS1-R [59]. Expectedly, our comparisons clearly show that our results are representative of a plant response to *Rs*.

Finally, given the expression profile of *CML8* in the roots, we compared the DEGs list with sets of genes specifically regulated in different root tissues [60,61,62]. Even though overlaps between lists were not significant, some genes were also found to be specifically expressed in the root cortex, pericycle, phloem pericycle, phloem companion cells, quiescent center, and endodermis [63]. Not surprisingly, these results also support that some genes identified in our study are involved in root-specific processes (Supplementary Tables S2–S4) and were consistent with the ones obtained using the Genevestigator database, where a small correspondence with abiotic stress studies and no overall tendency towards the developmental studies was detected (Supplementary Materials, Figure S5).

### 2.7. CML8 also Participates in Other Foliar and Root Micro-Organisms Plant Defense Responses

**Figure 6:**
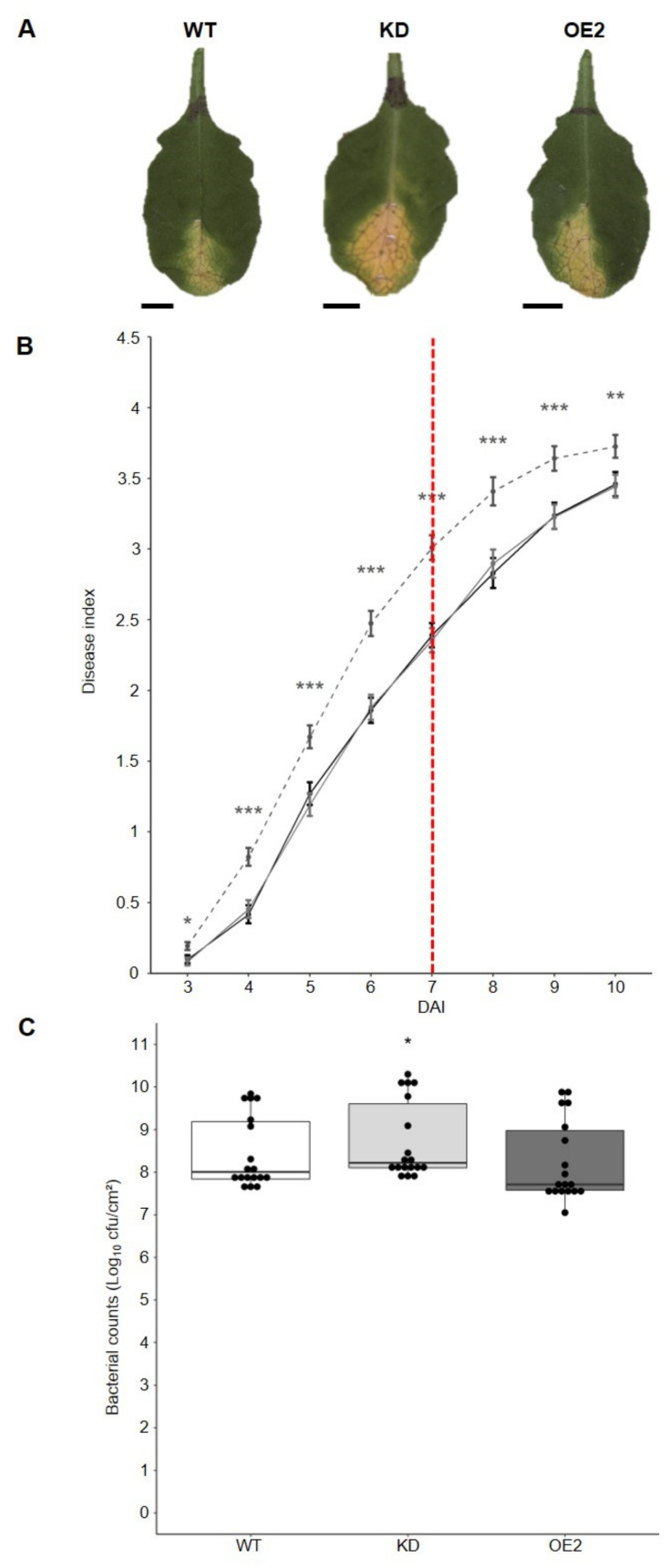
Comparative analysis of the susceptibility of *CML8* genotypes upon *Xcc* inoculation. (**A**) Photographs of disease symptoms were taken seven days after inoculation and represent the genotype’s average response to the inoculation. Bars: 0.5cm. (**B**) *Xcc* strain 8004 Δ*xopAC* (10^8^ cfu·mL^-1^) was inoculated by piercing onto *CML8* transgenic lines leaves. Disease index was scored from three to ten days after inoculation. Error bars = SEM were obtained from at least 24 inoculated leaves from six plants in four independent biological replicates. The red dotted line indicates the day when bacteria *in planta* quantification was performed. The solid black line, the dashed grey line and the solid grey line represent WT, *CML8* KD and OE2 lines, respectively. (**C**) Quantification of *in planta* bacterial growth was performed seven dai of *Xcc* strain 8004 Δ*xopAC* infection for WT, *CML8* KD and OE2 lines. Statistical analyses were performed as described in Materials and Methods section and significant difference was found with *p*-values < 0.05 (*** < 0.001, ** < 0.01, * < 0.05).

As shown here, and by Zhu et al. [26], CML8 is involved in the defense responses against two different types of bacteria, *Rs* and *Pst*, respectively, that have different lifestyles and infection modes. Considering the transcriptomic data and to determine if CML8 could be a common regulator in plant immune responses against various pathogens, the *CML8* genotypes were challenged by the aerial vascular biotrophic bacterium *Xcc* and the soil hemibiotrophic oomycete *Pc*. After inoculation with *Xcc* Δ*xopAC* strain, the disease symptoms (Figure 6A,B) appeared more rapidly in the *CML8* KD genotype compared to WT plants and *CML8* OE2 line as it was already observed following *Pst* [26] and *Rs* (Figure 3B) inoculations. In planta bacteria quantification clearly showed that bacteria growth was significantly higher in KD compared to WT (Figure 6C), indicating that CML8 acts as a positive regulator of defense responses following *Xcc* infection.

RT-qPCR experiments showed that the expression of *CML8* was slightly, but not significantly, induced (1.59-fold) at 24 hpi (Supplementary Materials, Figure S6A). However, with GUS staining of infected leaves, *CML8* was shown to be expressed from 6 h after *Xcc* inoculation (Supplementary Materials, Figure S7). *PDC1* and *JAL23,* which were respectively down- and upregulated in *CML8* KD plants at 24 hpi following *Rs* inoculation, had a similar expression profile in response to *Xcc,* even if the difference observed was not significant. However, for all other induced genes in OE2 line in response to *Rs* (*XBAT34, RLP41, LTP4-4, PNA-A,* and *NUDT6*), a reverse pattern was found (Supplementary Materials, Figure S6B).

**Figure 7:**
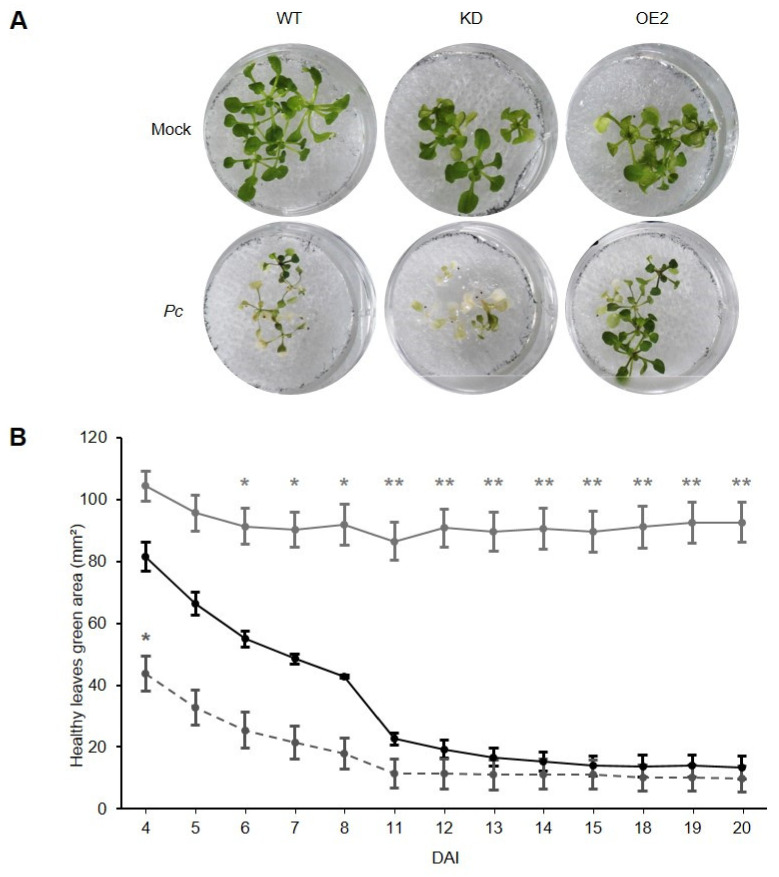
Comparative analysis of the susceptibility of *CML8* genotypes upon *Pc* infection. Two weeks-old seedlings grown on liquid MS medium were inoculated with 10^3^ zoospores from *Pc* LT3112 strain. (**A**) Photos represent the symptoms obtained at ten dai. (**B**) Quantification of disease was achieved by measuring the surface of green leaf area of inoculated plants from four to 20 days after inoculation. The solid black line, the dashed grey line and the solid grey line represent WT, *CML8* KD and OE2 lines, respectively. The data presented correspond to three independent replicates. Bars = SEM. Statistical analyses were performed as described in Materials and Methods section and significant difference was found with *p*-values < 0.05 (** < 0.01, * < 0.05).

Following inoculation of *CML8* genotypes with the oomycete *Pc*, disease quantification was realized by measuring the average surface of green tissue per seedling 10 days after treatment (Figure 7A,B). *CML8* KD and OE2 transgenic lines display, respectively, less and more green areas than the WT, suggesting that CML8 also contributes to a better tolerance against *Pc.*

At gene expression level, using RT-qPCR and GUS activity, *CML8* transcription was not modified following *Pc* infection (Supplementary Materials, Figure S6C, Figure S8). *JAL23*, *XBAT34*, *LTP4.4,* and *RLP41* showed a reverse expression pattern after *Pc* inoculation when compared to *Rs* inoculation. Interestingly, *NUDT6* was significantly induced in OE2 line at 24 hpi in both cases of *Pc* and *Rs* inoculations. Although not significant, two other genes (*PDC1* and *PNP-A*) showed the same tendency during *Pc* and *Rs* inoculations, as they were respectively down- and upregulated in both instances (Supplementary Materials, Figure S6D). Taken together, these results suggest that CML8 acts as a regulator to multiple pathogens, probably by controlling common and specific immune processes following attacks by vascular root and leaf bacteria, as well as soilborne oomycetes.

## 3. Discussion

The sequencing of the Arabidopsis genome, as well as many other plant genomes since 2000, has shown the existence in plants of a large number of Ca^2+^ sensors [21,64,65,66,67]. Indeed, it was shown that the number of genes encoding the typical CaM, as well as the related CMLs, is high, ranging from 1 CaM and 3 CMLs in the green algae *Ostreococcus lucimarinus* to 6 CaM and 56 CMLs in poplar [21]. This raises the question about their respective roles in plant physiology. Concerning CML8, previous works indicate that CML8 binds Ca^2+^, controls enzyme activity, and is involved in *Pst* defense responses as a positive regulator [26,68,69]. Here, we show that CML8, a root- and vascular tissues-expressed CML, participates in defense responses not only against the soilborne bacterium *Rs,* but also to other pathogens, such as the epiphytic vascular bacterium *Xcc* and the oomycete *Pc*. Plant susceptibility increased in response to *Xcc* and *Pc,* and this phenotype was correlated with a greater in planta bacterial multiplication for *Xcc*. Complementarily, *CML8* OE lines were less susceptible and repressed bacterial multiplication in response to *Rs,* supporting that CML8 acts as an activator of defense responses. To better understand the molecular processes that can be under the direct or indirect control of CML8, an RNA-seq approach focused on the identification of early root-regulated genes by *Rs* infection allowed to identify 151 DEGs. Noteworthy, when unchallenged by *Rs*, very few genes were differentially regulated in the *CML8* transgenic lines. Only eight and two DEGs were identified in KD and OE2, respectively, and none of these genes were present in the list of 151 DEGs, suggesting that CML8 may act as a sensor tightly regulated by the plant to rapidly cope with external stimuli such as interaction with pathogens at the root level during development. Surprisingly, the number of genes whose transcriptional regulation is CML8-dependent was small compared to the importance of the late phenotypic response of the transgenic plants following inoculation, whatever the pathogen used. This could be explained by the experimental design setup to identify the molecular actors involved in the early response, by the statistical model used for the analysis of the RNA-seq data and the selected interaction, by the nature of the tissue analyzed, and, in particular, by the spatiotemporal regulation of *CML8* expression. Therefore, we suggest these DEGs mainly correspond to a finely tuned and localized induction of a plant immune response sufficient to allow the plants to resist or to cause a delay in the development of the diseases. Interestingly, while *CML8* appears to be weakly expressed in leaves [26], its specific transcriptional regulation in the stomatal guard cell appears also in accordance with its role as a positive regulator of the plant defense response to *Pst*, stomata being important entry sites in host tissue for the efficient infection by this pathogenic bacteria [70]. Consistently, among our list, numerous genes are known to be regulated by several and various pathogens, and are mostly involved in basal defense. Indeed, defense-related genes, such as *PR2*, SA biosynthesis, and signaling genes (*PAD4*, *EDS1*, *EDS5*, *EDS16*), and camalexin synthesis gene *PAD3,* were all found significantly induced in OE2 plants compared with WT after *Rs* inoculation. In addition, PR1 protein accumulation was higher in OE plants and lower in KD plants compared to WT plants, and SA content was significantly lower in KD plants compared to WT plants during an inoculation with *Pst* [26].

Among the most promising candidates of our RNA-seq data, two cell wall-related genes strongly induced in *CML8* OE2 line were identified: *PNP-A*, already shown to play a role in host defense against pathogens [35], and the lipid transfer protein *LTP4.4* gene, crucial in the resistance against *Fusarium* trichothecene mycotoxin [34]. These two genes belong to the same co-expression network, along with other highly induced DEGs in *CML8* OE2 line: *RLP41*, *NUDT6,* and *XBAT34*. During plant infection by soil pathogens, primary metabolism, such as ethanol fermentation, is altered [38]. The pyruvate decarboxylase *PDC1* and the alcohol dehydrogenase *ADH1* genes are both strongly repressed in *Rs*-infected roots of *CML8* KD plants. Interestingly, these two genes are always found together in co-expression networks, and proteins are predicted to physically interact. However, as *ADH1* was also strongly downregulated in *CML8* OE2 plants, it may be that only *PDC1* plays a role in plant host immunity to *Ralstonia*. These genes have been shown to be induced in the early stages of Arabidopsis infection by the causal agent of clubroot *Plasmodiophora brassicae* [71], and are necessary for plant survival under hypoxic conditions [72]. This may indicate that CML8-mediated signaling is required for PDC1 induction during root response to nonoptimal conditions, related to stresses of biotic or abiotic nature. Another family of proteins that is important in plant response to various stresses is the Jacalin lectin family, by binding carbohydrate ligands, thus perceiving many environmental signals [39]. For example, Weidenbach et al. [73] showed that the Jacalin-related lectin domain containing rice protein JAC1 confers quantitative resistance to bacteria, oomycetes, and fungi when overexpressed. Three proteins of this family were induced in KD plants at 24 hpi, and *JAL23* was the most strongly regulated. Unlike during *Rs* bacterial infection, only two and three of these genes were similarly regulated during *Xcc* and *Pc* infections, respectively. This could be explained by the differences in infected tissue, *Xcc* being a leaf pathogen, and type of microorganism, *Pc* being an oomycete.

These data support that CML8 does not present a total functional redundancy with the other CMLs [25,26,74,75]. This could be explained by differences in their expression profiles but also to their downstream target repertoire, which are most likely different. Indeed, *CML8* is mainly transcribed in roots, and particularly during LR formation at primordia level, but also at a low level in leaf vascular tissues. This expression pattern strengthened following *Rs* inoculation, *CML8* expression being rapidly and transiently induced in both root and leaf vascular tissues where *Rs* propagates and multiplies. Consistent with results previously obtained with *Pst* [26], *CML8* expression is also slightly induced and spreads throughout the vascular system near the site of infection in response to *Xcc* (Supplementary Materials, Figure S7). As previously shown, *CML8* gene expression is not positively or negatively regulated by PAMPs such as flagellin and Ef-Tu [26]. Knowing that hormones are involved in plant development but also in plant immune responses [76], we explored their putative effect on the regulation of *CML8* spatiotemporal expression. *CML8* gene expression patterns were analyzed following exogenous hormones treatment. Results indicated that *CML8* could be induced by ethylene, SA, GA, and brassinosteroids. SA has long been known to promote plant defenses against many pathogens, including *Xcc* [77], *Pc* [78], and *Pst* [76]. *Rs* even possesses an SA degradation pathway to decrease its toxicity [79]. It is worth noting that many pathogens are able to produce certain phytohormones to manipulate plant defenses, as it has been shown for *Rs*, which can produce ethylene [80]. Indeed, we show that *CML8* is expressed in LRs where the bacterium enters the host and is induced in the presence of *Rs*. Thus, we cannot exclude that *Rs* can control the expression of *CML8* to promote its infection by hormones such as ethylene [80].

CML8 was not the first CML identified as a regulator of defense against *Rs* infection. Indeed, Shen et al. [81] demonstrated that the overexpression of *CML13* from pepper induces hypersensitive reaction, whereas its silencing triggers plant susceptibility. Zheng et al. [82] showed that *Rs* interferes with Ca^2+^-dependent gene expression to promote disease development in potato. The RipAB effector is responsible for the repression of three *CMLs*, one *CDPK,* and a Ca^2+^ transporter in potato. In 2021, Meng et al. [83] reported that among the root genes, specifically, and rapidly upregulated in tobacco-resistant cultivar to *Phytophthora nicotianae*, four *CMLs* were identified, whereas the *Loc107773369* gene, which corresponds to the closest tobacco ortholog to *CML8*, was downregulated. CML8 is the closest homolog to the soybean CaM4 and when overexpressed, CaM4 is responsible for enhanced resistance to the oomycete *Phytophthora sojae* and two necrotrophic fungi, *Alternaria tenuissima* and *Phomopsis longicolla* [84]. Still, few examples are reported for a gene able to confer plant multipathogen resistance. As an example, the tomato immune receptor Roq1 confers immunity to *Xcc*, *Pst,* and *Rs* by inhibiting pathogen virulence and activating at least two independent downstream defense responses [85]. Altogether, our results support that CML8 acts in a signaling hub required for the establishment of basal defense responses to a wide variety of plant pathogens, probably through hormones interconnected signaling pathways, rather than PAMP pathways as reported in Zhu et al. [26].

Many CMLs have been reported to be involved in both biotic and abiotic stress responses. Interestingly, CML8 appears to be one of the rare CMLs reported to date, involved in plant defense responses to several pathogen species. These data illustrate the complexity of Ca^2+^ signaling in biotic and abiotic responses and highlight the importance of Ca^2+^ in such signaling. Increasing papers have shown that environmental changes such as elevated temperatures negatively impact a majority of resistance response to many pathogens attack [2]. Aoun et al. [86] showed that the *Rs* resistance response linked to the RPS4/RRS1-R immunoreceptor pair, effective at 27 °C, is inhibited at 30 °C. Hilleary et al. [87] also demonstrated that, following flagellin treatment on Arabidopsis, Ca^2+^ signals are altered when the temperature increases from 22 °C to 28 °C. The next step will be to investigate the robustness of CML8-dependent immune responses in contrasted abiotic and/or biotic environments and to identify the molecular actors differentially regulated by CML8.

## 4. Materials and Methods

### 4.1. Plant Materials and Culture Conditions

*Arabidopsis thaliana* Columbia Col-8 plant corresponding to the wild type (WT) genetic background was used as control. The knockdown *amiRNA 2.3.2* (KD) and *CML8* overexpressing transgenic Arabidopsis lines *OECML8 2.3* (OE2) and *OECML8 3.2* (OE3), have been already characterized in our previous work [26]. To avoid variations in seed quality, seeds were produced from plants cultivated over the same time period and stored in identical conditions. Seeds were surfaced sterilized as described by Zhu et al. [26].

### 4.2. Bacteria and Oomycete Strains and Growth Conditions

The *Rs* WT reference GMI1000 strain was used for RNA-seq experiments. For symptom notations and IGC, the *Rs* mutant strain Δ*popP2* complemented with PopP2 (strain GRS100) that behaves similarly to GMI1000 [88] was used as control to investigate a potential role of RRS1 in CML8-mediated responses. Both strains were grown at 28 °C in BG medium as described by Plener et al. [89]. The *Xanthomonas campestris* pv. *campestris* (*Xcc*) strains were the 8004 Δ*xopAC* strain that is deleted for the avirulence gene *xopAC* [90] and the strain Δ*xopAC*-GUS-GFP that constitutively expresses *uidA* and *gfp* genes [91]. *Xcc* was cultivated in MOKA medium [92]. The LT3112 *Phytophtora capsici* (*Pc*) strain used was grown in a controlled culture chamber at 22 °C on V-8 agar medium [93].

### 4.3. Plant Inoculations and in Planta Quantifications

*Rs* inoculations were performed on four-week-old plants using the method described in Aoun et al. [86]. To promote bacterial entry and obtain a homogeneous infection, and to better assess the magnitude of the effect of CML8 on plant immunity, roots were cut, and plants were soaked for 15 min in 2 L per tray of a bacterial suspension at 1 × 10^7^ cfu mL^−1^ for symptom notations and IGC and transferred in growth chambers with controlled 27 °C light/26 °C dark conditions (75% relative humidity, 12 h light, 100 μmol m^−2^ s^−1^). The wilting symptoms were scored on an established zero, one, two, three, and four disease index scale corresponding to healthy, 25%, 50%, and 75% wilted and dead plants, respectively. Data in Figure 3 are representative of three independent experiments consisting of 69 plants per genotype. Data in Supplementary Materials, Figure S2 are representative of three independent experiments consisting of 75 and 73 plants for GMI1000 and GRS100 inoculations, respectively. Quantification of bacteria in planta was performed as described in Deslandes et al. [94]. For *Xcc*, piercing inoculations were carried out on four-week-old plants as described by Meyer et al. [95], and disease index scoring varies from zero (no symptom) to four (leaf death). In planta bacteria quantifications were performed after seven days as described by Xu et al. [96]. For *Pc*, inoculations and symptoms quantifications were performed as described by Larroque et al. [93]. Symptoms were quantified from photos with ImageJ software (ImageJ. Available online: https://imagej.nih.gov/ij/, accessed time: 25 September 2021); the leaf surface of green area was used as proxy for disease development. The WT accession was used as a control in all experiments, and bacteria quantification experiments were performed at least three times using six leaves from six independent plants for *Xcc* and three aerial parts of three independent plants for *Rs*.

### 4.4. Analyses of the CML8 Gene Expression Pattern in Transgenic Seedlings and in Response to Rs, Xcc, and Pc Inoculations and Exogenous Hormones Treatment

Homozygous transgenic plants harboring the *CML8 promoter::uidA* construct [26] were used to monitor the *CML8* gene expression pattern either in control conditions or following *Rs*, *Xcc*, and *Pc* inoculations. *Xcc* and *Pc* inoculations were performed as described above. For *Rs*, *CML8 promoter::uidA* transgenic Arabidopsis seedlings were grown for eight days in liquid MS medium (0.5×, pH 5.7, 1% sucrose) in 24-well plates. *Rs* inoculations were carried out by replacing the MS medium with 1 × 10^7^ cfu·mL^−1^ *Rs* or not (mock) in growth chambers at 27 °C for six hours before GUS staining. For *CML8* expression pattern in response to hormones, 8-day-old seedlings of *promoter CML8::uidA* transgenic lines were transferred to liquid MS with or without hormones for 3, 6, and 24 h. Hormones used were 1-Aminocyclopropane-1-carboxylic acid (ACC, ethylene precursor 10 µM), brassinosteroid (BR at 100 nM), salicylic Acid (SA 100 µM), gibberellic acid (GA 10 µM), abscisic acid (ABA 10 µM), methyl jasmonate (MJ at 10 µM), GR24 (strigolactone analog at 15 µM), and Auxin (IAA 1 µM). GUS staining was performed as described by Magnan et al. [24], and pictures were taken using Axio Zoom v16 (Zeiss, Kelsterbach, Germany).

### 4.5. Global Transcriptome Analyses Using ILLUMINA RNA-seq

Four-week-old WT, *CML8* KD, and OE plants were root-inoculated without cutting the roots, as described in Aoun et al. [86], either with water (mock) or *Rs* GMI1000 strain. Roots from mock- and *Rs*-inoculated plants were harvested after 0, 6, 12, and 24 hpi. Each sample is composed of the roots of five plants. For each sample, three independent biological replicates were generated. RNA were extracted using a CTAB protocol [97] and treated with DNase (Thermo Fisher Scientific Life Science, Waltham, USA), and RNA integrity was assessed using the Agilent RNA 6000 nano kit and the Agilent 2100 Bioanalyzer system. Four µg RNA per sample was used as input material for the RNA sample preparations. Sequencing libraries were generated using NEBNext^®^ UltraTM RNA Library Prep Kit for Illumina^®^ (NEB, Ipswich, USA) and sequences index codes were attributed to each sample. The clustering of the index-coded samples was performed on a cBot Cluster Generation System using TruSeq PE Cluster Kit v3-cBot-HS (Illumina, San Diego, USA). After cluster generation, the libraries were sequenced on an Illumina Novaseq platform and 150 bp paired-end reads were generated. Oriented paired-end RNA sequencing was carried out by Novogene Bioinformatics Technology Co., Ltd. (Tianjing, China). Four GB raw data were obtained for each sample library. Clean reads were obtained by removing adapter and reads containing poly-N, and low-quality reads were eliminated. Raw sequence data were submitted to the Sequence Read Archive (SRA) database (Accession SRP280329).

### 4.6. Statistical Analysis of RNA-seq Data

Pair-end reads from the 63 RNA-seq runs were aligned on the Arabidopsis TAIR10 genome with tophat-2.1.1. Read countings were performed with htseq-0.9.1. An average of 36 million reads with quality scores over 90% per sample were obtained. To identify DEGs, htseq-counts files were analyzed with the R software, also using EdgeR package version 3.24.3 [98]. A genotype comparison between treated and nontreated plants at a given time point was performed. Genes that did not have at least one read after a count per million normalization in at least one half of the samples were discarded. Raw counts were normalized using TMM method and count distribution was modeled with a negative binomial generalized linear model where the genotype, the treatment, the time, and all double interactions between factors were taken into consideration, and dispersion was estimated by the edgeR method. A likelihood ratio test was performed to evaluate an infection effect in a genotype at a given time point. Raw *p*-values were adjusted with the Benjamini–Hochberg procedure to control the false discovery rate (FDR). A gene was declared differentially expressed if its adjusted *p*-value was ≤0.05. A list of DEGs was recovered for both KD vs WT and overexpressor OE vs WT comparisons, based on a 5% FDR correction (Supplementary Materials, Table S1).

### 4.7. Biological Pathway Enrichment, Gene Network Analysis, Comparisons with Available Transcriptome Datasets

Biological pathways significantly overrepresented were identified with the Classification SuperViewer tool using MapMan classification categories (The Bio-Analytic Resource for Plant Biology. Available online: http://bar.utoronto.ca/ntools/cgi-bin/ntools_classification_superviewer.cgi, accessed time: 25 September 2021) [99]. We used GeneMANIA to predict the contribution of DEGs in co-expression or interaction networks (https://genemania.org/, accessed time: 25 September 2021) [100]. To infer the putative function of DEGs, hierarchical clustering analyses were performed using the Genevestigator toolbox (Genevestigator. Available online: https://www.genevestigator.com/gv/, accessed time: 25 September 2021) and microarrays and RNA-seq data coming from stress, biotic, elicitor, and development data, with distance measured as Euclidian distance.

Lists of genes were also compared to specific lists of Arabidopsis genes involved in Ca^2+^ signaling [45], auxin [101], root development [60,61,62,63], and genes differentially regulated during PTI, ETI, or both in response to different pathogens [43,47,48,49,50,51,52,53,54,102,103,104] including *Rs* [55,56,57,58,59,105]. The statistical enrichment of DEGs in these lists was evaluated using a hypergeometric statistic test (*p*-value ≤ 0.05) (R version 3.5.2 (2018-12-20)).

### 4.8. Statistical Analysis of Phenotyping and Bacteria Quantifications

For each transgenic of phenotyping experiments for *Rs* and *Xcc* inoculations, the following mixed model was used to test whether the transgenic plant differed from the WT background:

disease index ij = µ + block i + genotype j + block i × genotype j + ε ij(1)

where µ is the overall mean of the phenotypic data, “block” accounts for differences in microenvironmental conditions between the experimental blocks, “genotype” corresponds to the genetic differences between the transgenic and the wild-type background, “block × genotype” accounts for variation in between genotype differences among blocks, and “ε” is the residual term. All factors were considered as fixed. Model (1) was applied separately to each transgenic and the corresponding background. This model was implemented with the function lm() in R software environment (R version 3.6.1 (2019-07-05) R Core Team 2019). The dynamics of mutant responses to *Rs* and *Xcc* were presented using the ggplot2 package [106] showing the least-square means (LSmeans) ± the standard error of the LSmeans. For phenotyping experiments for *Pc* inoculations, data were analyzed using variance analyses performed using the version 9.4 of SAS software. To analyze bacteria quantifications in planta, each transgenic plant was analyzed with the following mixed model to test whether the transgenic differed from the WT background:

number of CFU/FW (g) ij = μ + block i + genotype j + block i × genotype j + ε ij(2)

where μ is the overall mean of the phenotypic data, “block” accounts for differences in microenvironmental conditions between the experimental blocks, “genotype” corresponds to the genetic differences between the transgenics and the wild-type background, “block x genotype” accounts for variation in between genotype differences among blocks, and “ε” is the residual term. All factors were considered as fixed. Model (2) was applied separately to each pair of transgenic plant and its corresponding WT. This model was implemented with the function lm() in R software environment. The log of cfu per gram of fresh weight averaged over the two plates for *Rs* inoculations and the log of cfu per cm^2^ for *Xcc* inoculations were represented as a boxplot per genotype using the ggplot2 package [106]. Error bars represent standard error means (SEM) (R version 3.6.1 (2019-07-05) R Core Team 2019).

### 4.9. RT-qPCR Experiments

Total RNA from two biological replicates of *Rs*-inoculated roots were used for *CML8* expression. Total RNA was prepared from four leaves from four independent plants inoculated at zero and 24 hpi by piercing with *Xcc* and from roots of 15 independent seedlings inoculated at zero and 24 hpi with spores of *Pc* using the EZNA Plant RNA Kit (Omega Bio-tek^®^ R6827-02, Norcross, GA, USA). For each experiment, two independent biological replicates and two technical replicates were performed. Totals of 500 ng to 1 µg of total RNA were treated with DNAse (Ambion AM1907, Thermo Fisher Scientific Life Science, Waltham, MA, USA) and reverse-transcribed (Thermo Fisher Scientific Life Science, Waltham, MA, USA) with RNase inhibitor (Applied Biosystem, Thermo Fisher Scientific Life Science, Waltham, MA, USA). qPCR was performed with cDNA diluted 1/10th or 1/20th on a QuantStudio 6 Real-Time PCR Systems (Thermo Fisher Scientific Life Science, Waltham, MA, USA). *EF1-alpha* (*At5g60390*) was used for normalization, and the ^ΔΔ^ Ct method [107] was used to calculate fold changes relative to the internal control and the mock-inoculated control samples. Primer sequences used are listed in Supplementary Materials, Table S5. Student’s t-test was used, and significant difference was found with *p*-values < 0.05 (*** < 0.001, ** < 0.01, * < 0.05).

## Data Availability

The data presented in this study are available are openly available in SRA database (Accession SRP280329) and on request from the corresponding author.

## Supplementary Materials

The following are available online at https://www.mdpi.com/article/10.3390/ijms221910469/s1.
