# Supplementary material for: The Same against Many: AtCML8, a Ca2+ Sensor Acting as a Positive Regulator of Defense Responses against Several Plant Pathogens
Source: Int J Mol Sci. 2021 Sep 28;22(19):10469. doi: 10.3390/ijms221910469 (PMC8508799; doi:10.3390/ijms221910469)
Supplement: Supplementary file 1 [file ijms-22-10469-s001.zip › Figure S1.pdf]

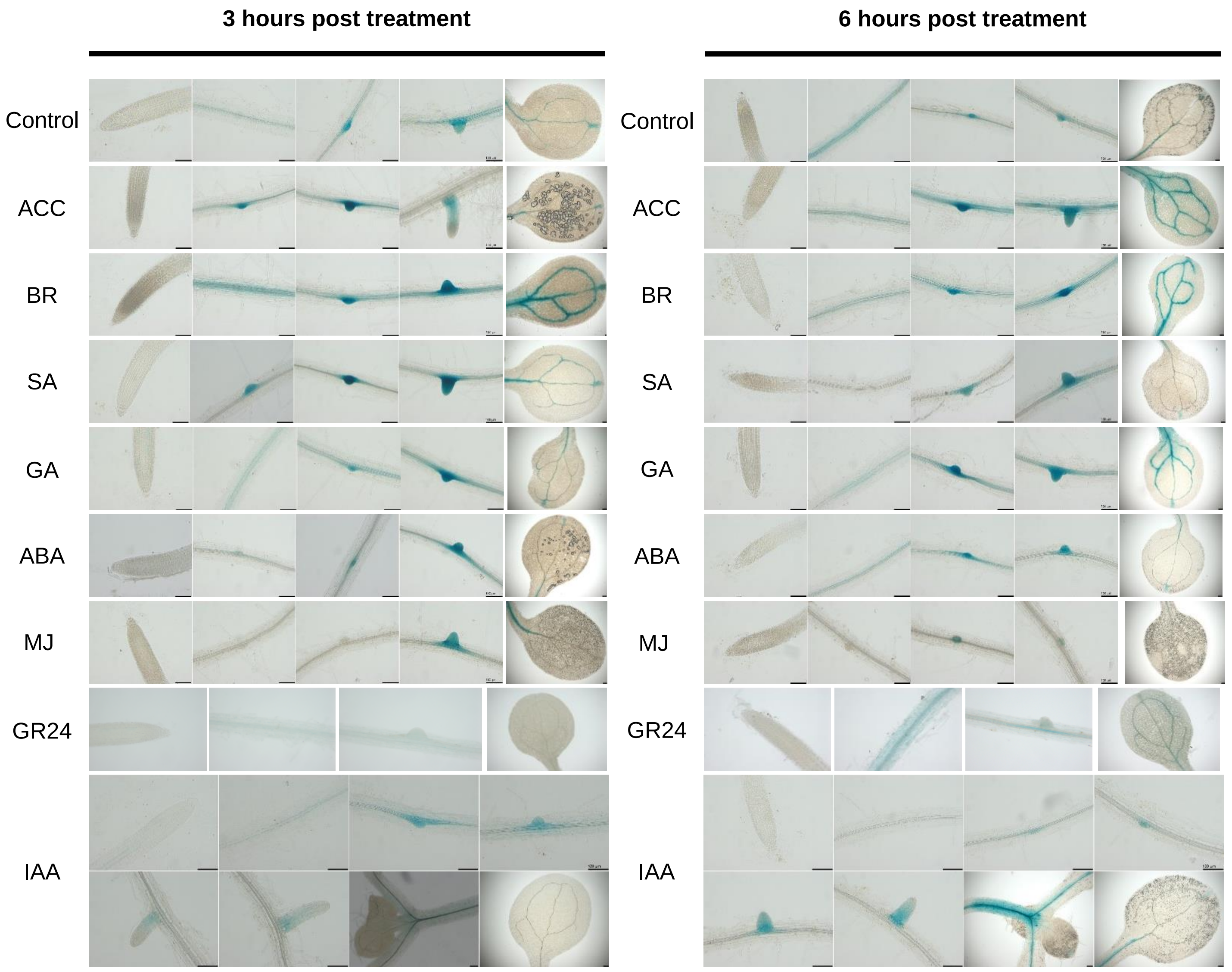
3 hours post treatment
6 hours post treatment
Control
ACC
BR
SA
GA
ABA
MJ
GR24
IAA

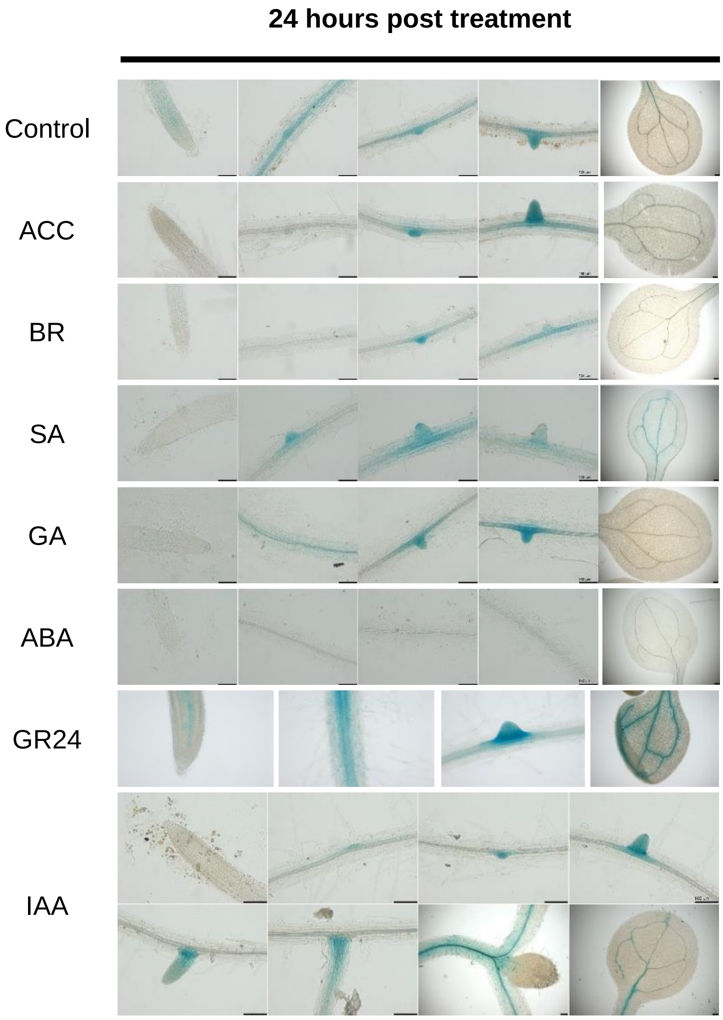

Figure S1. *CML8* expression pattern in response to hormones treatments. Eight days-old seedlings of promoter *CML8::uidA* transgenic lines were transferred to liquid MS with or without hormones for 3 hours, 6 hours and 24 hours before GUS staining. Hormones used are 1-Aminocyclopropane-1-carboxylic acid (ACC, ethylene precursor 10 µM), Brassinosteroids (BR, 100 nM), Salicylic Acid (SA, 100 µM), Gibberellic acid (GA, 10 µM), Abscisic acid (ABA, 10 µM), Methyl Jasmonate (MJ, 10 µM), GR24 (strigolactone analog, 15 µM) and Auxin (IAA, 1 µM).Bars: 100 µm.
