# Supplementary material for: The Same against Many: AtCML8, a Ca2+ Sensor Acting as a Positive Regulator of Defense Responses against Several Plant Pathogens
Source: Int J Mol Sci. 2021 Sep 28;22(19):10469. doi: 10.3390/ijms221910469 (PMC8508799; doi:10.3390/ijms221910469)
Supplement: Supplementary file 1 [file ijms-22-10469-s001.zip › Figure S2.pdf]

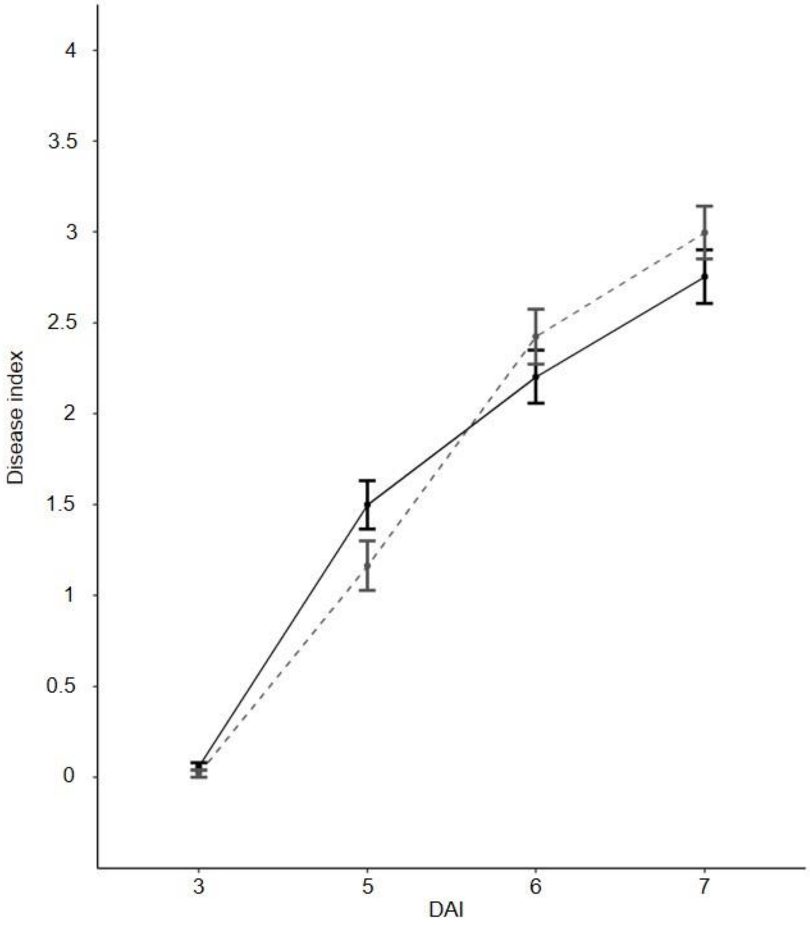

Figure S2. Comparative analysis of the susceptibility of Arabidopsis WT Col-8 lines upon inoculation with *Rs* GMI1000 WT strain and GRS100 strain that is deleted for the effector PopP2 complemented with PopP2. Cut roots of four weeks-old plants of WT with a suspension of 1.10^7 $cfu.mL^{-1}$ of *Rs* GRS100 strain. Disease symptoms index are shown from three dai to seven dai. The solid black line and the dashed grey line represent GMI1000 and GRS100 inoculations, respectively. Error bars = SEM were obtained from 75 and 73 plants for GMI1000 and GRS100 inoculations respectively, in three independent biological replicates.
