# Supplementary material for: The Same against Many: AtCML8, a Ca2+ Sensor Acting as a Positive Regulator of Defense Responses against Several Plant Pathogens
Source: Int J Mol Sci. 2021 Sep 28;22(19):10469. doi: 10.3390/ijms221910469 (PMC8508799; doi:10.3390/ijms221910469)
Supplement: Supplementary file 1 [file ijms-22-10469-s001.zip › Figure S4.pdf]

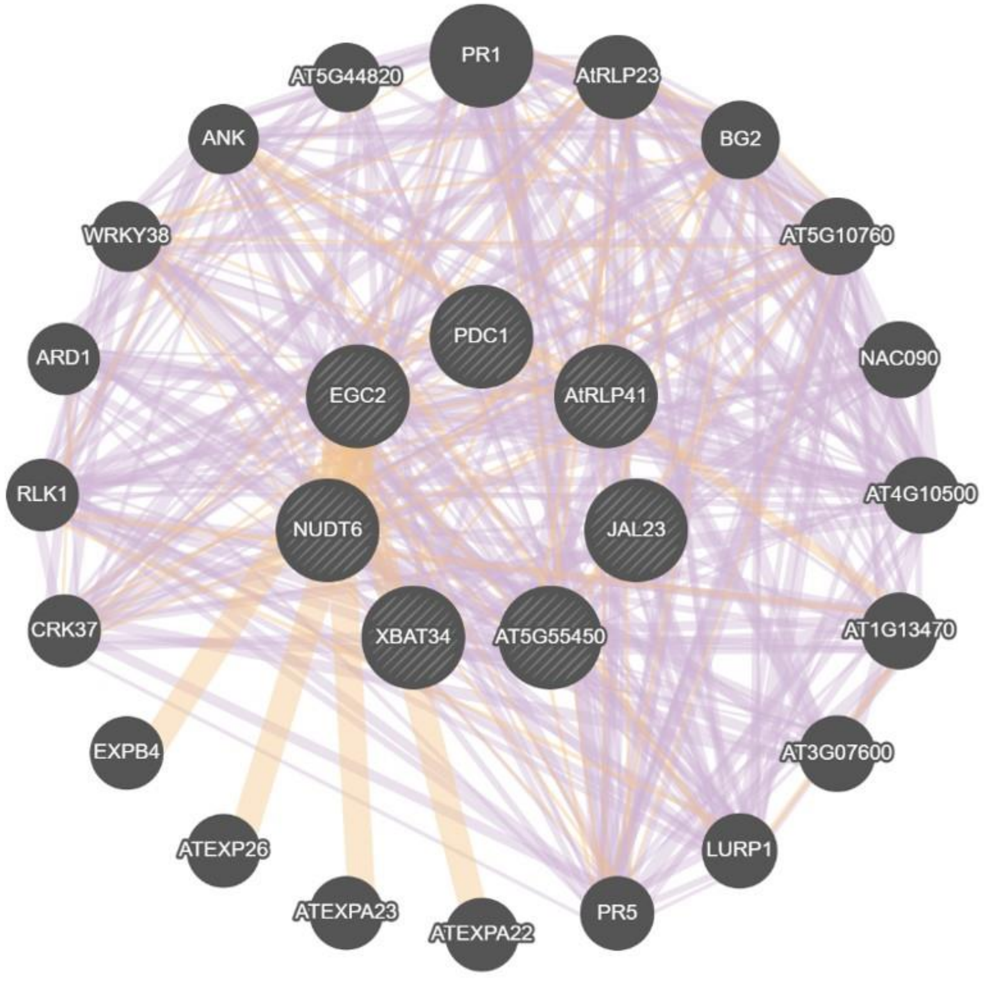

Figure S4. Co-expression network of seven candidate *CML8*-dependent DEGs (Supplementary Materials, Table S1) following *Rs* infection in KD and OE lines, obtained with GeneMANIA online tool. The purple lines represent co-expression relationships and the orange line represent predicted protein-protein interaction.
