# Supplementary material for: The Same against Many: AtCML8, a Ca2+ Sensor Acting as a Positive Regulator of Defense Responses against Several Plant Pathogens
Source: Int J Mol Sci. 2021 Sep 28;22(19):10469. doi: 10.3390/ijms221910469 (PMC8508799; doi:10.3390/ijms221910469)
Supplement: Supplementary file 1 [file ijms-22-10469-s001.zip › Figure S5.pdf]

A: Biotic selection with Microarray studies

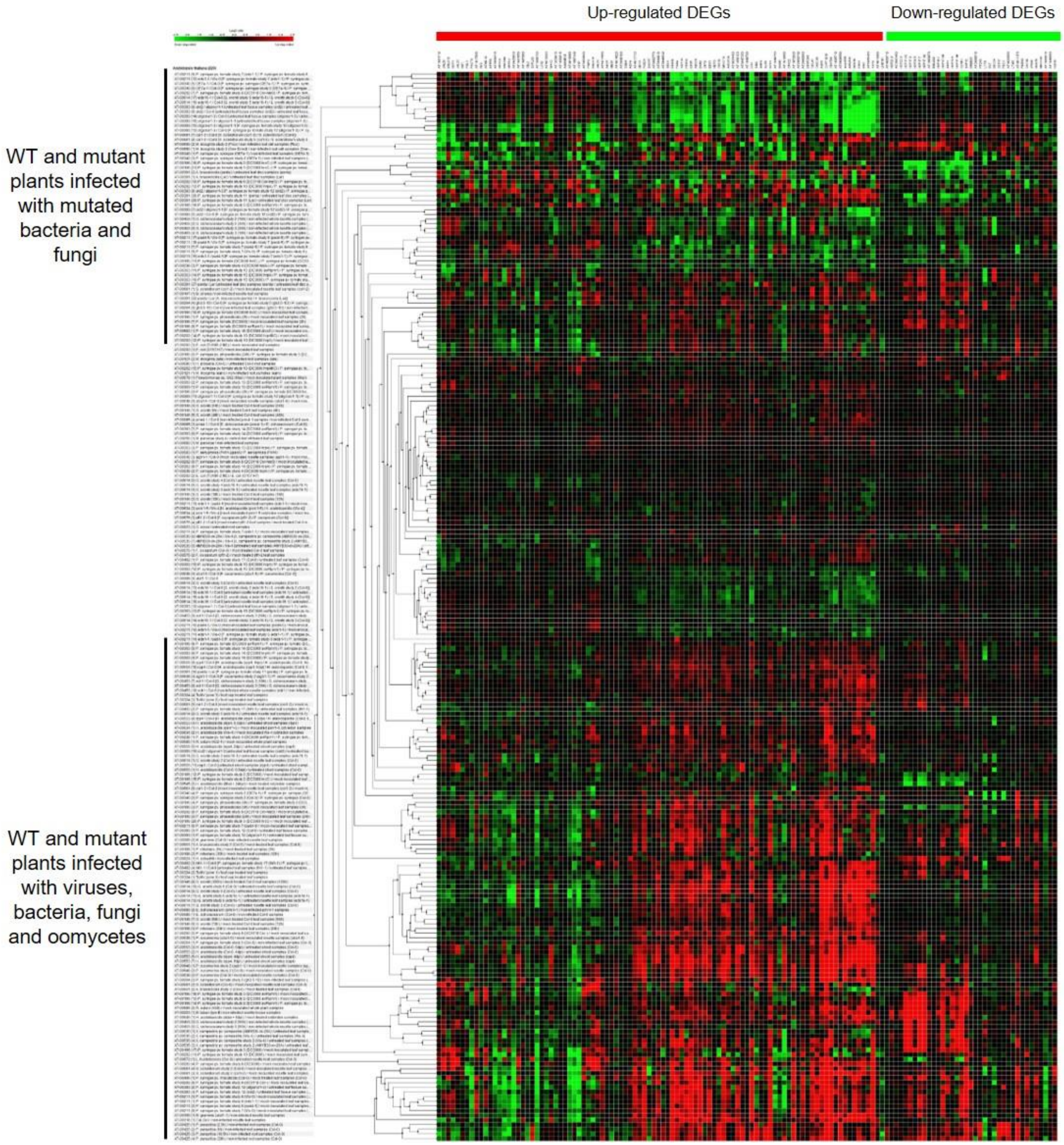

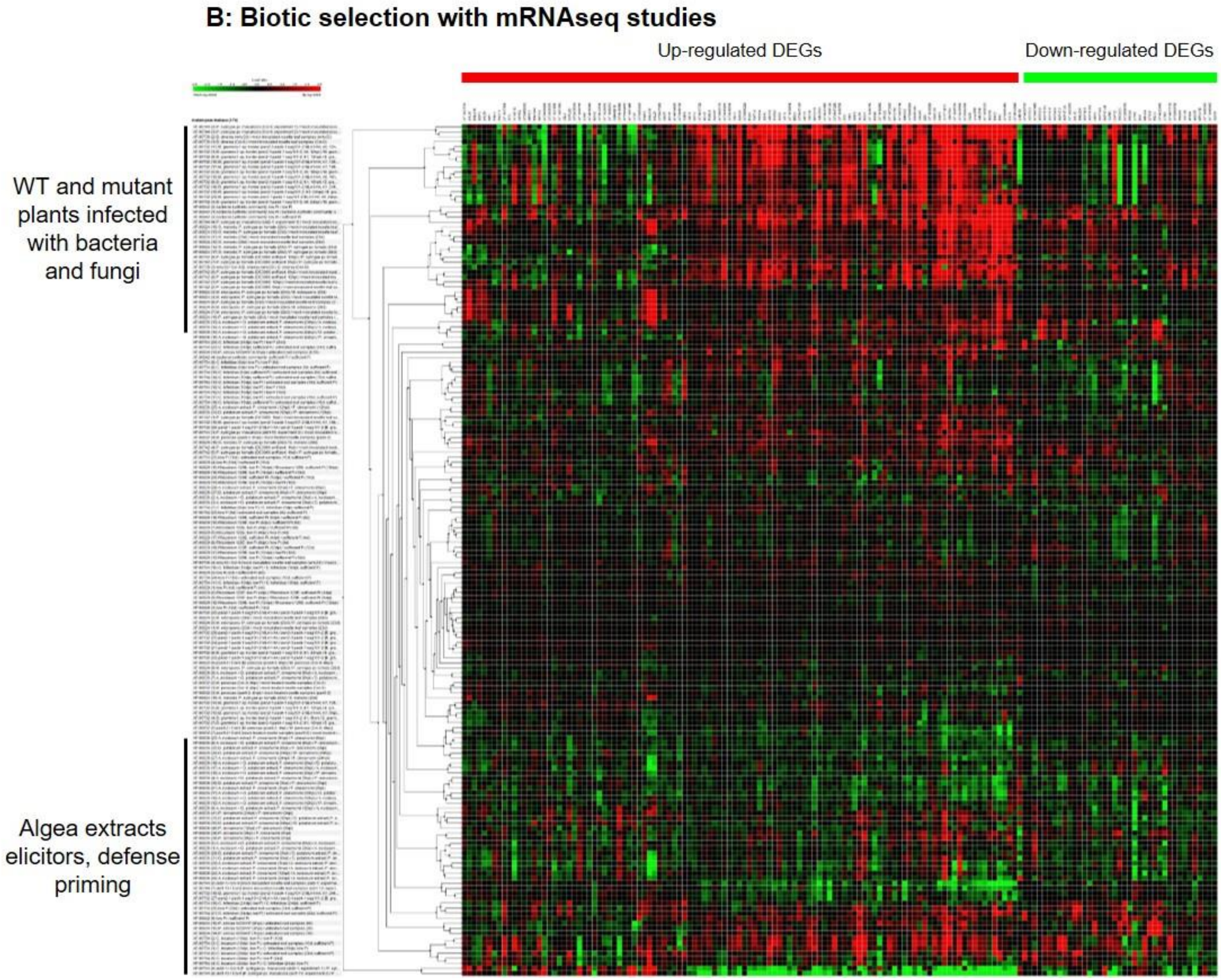
B: Biotic selection with mRNAseq studies
Up-regulated DEGs
Down-regulated DEGs
WT and mutant plants infected with bacteria and fungi
Algea extracts elicitors, defense priming

C: Elicitor selection with Microarray studies

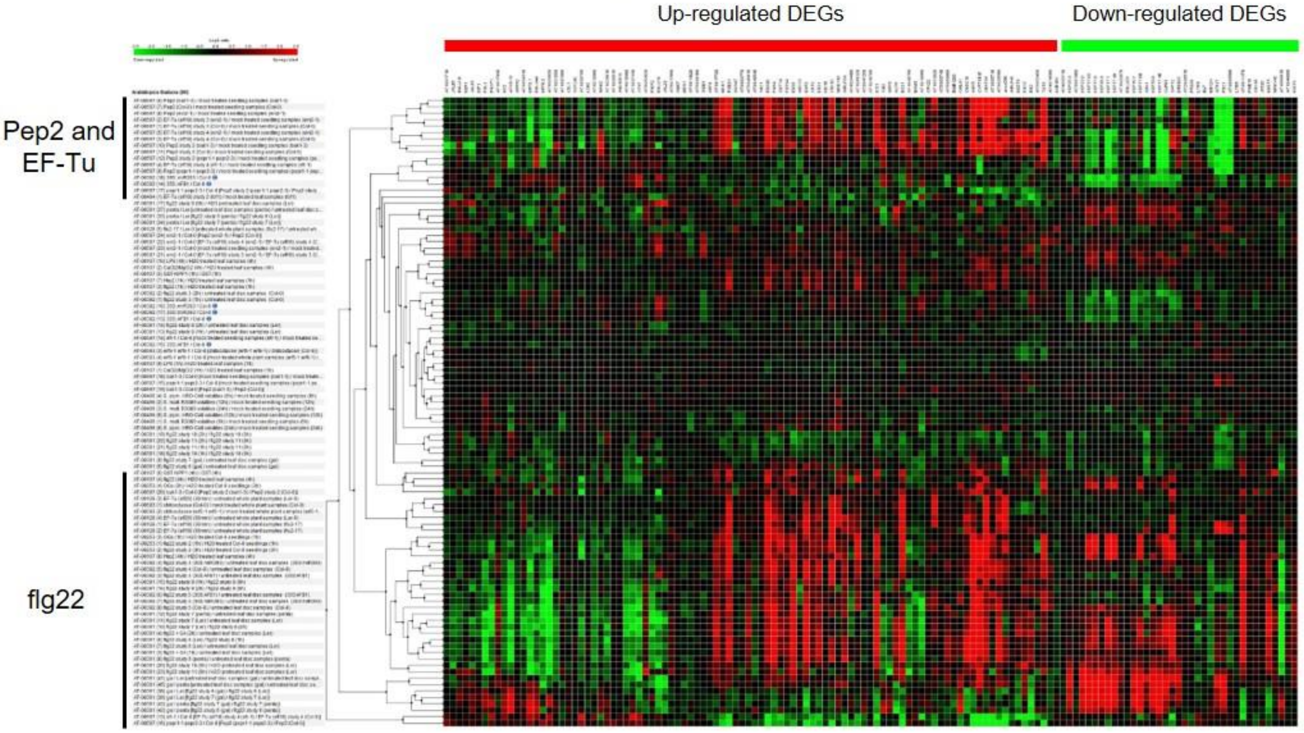

D: Elicitor selection with mRNAseq studies

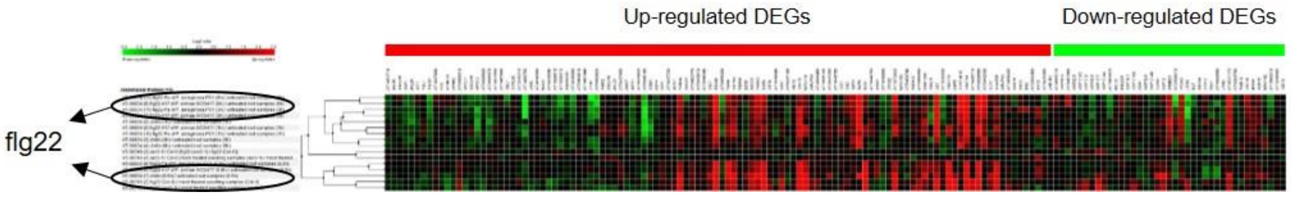

E: Stress selection with Microarray studies

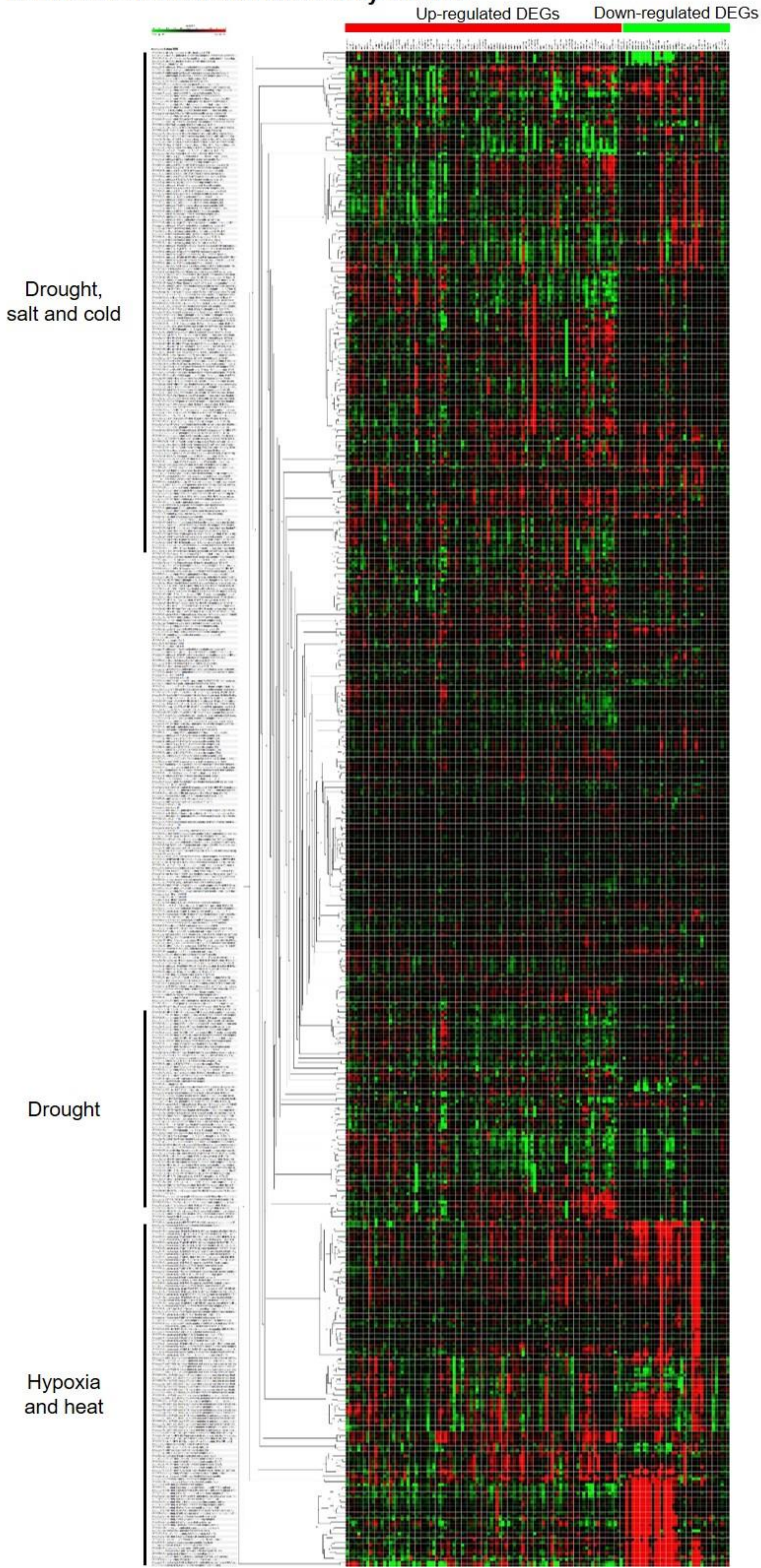

F: Stress selection with mRNAseq studies

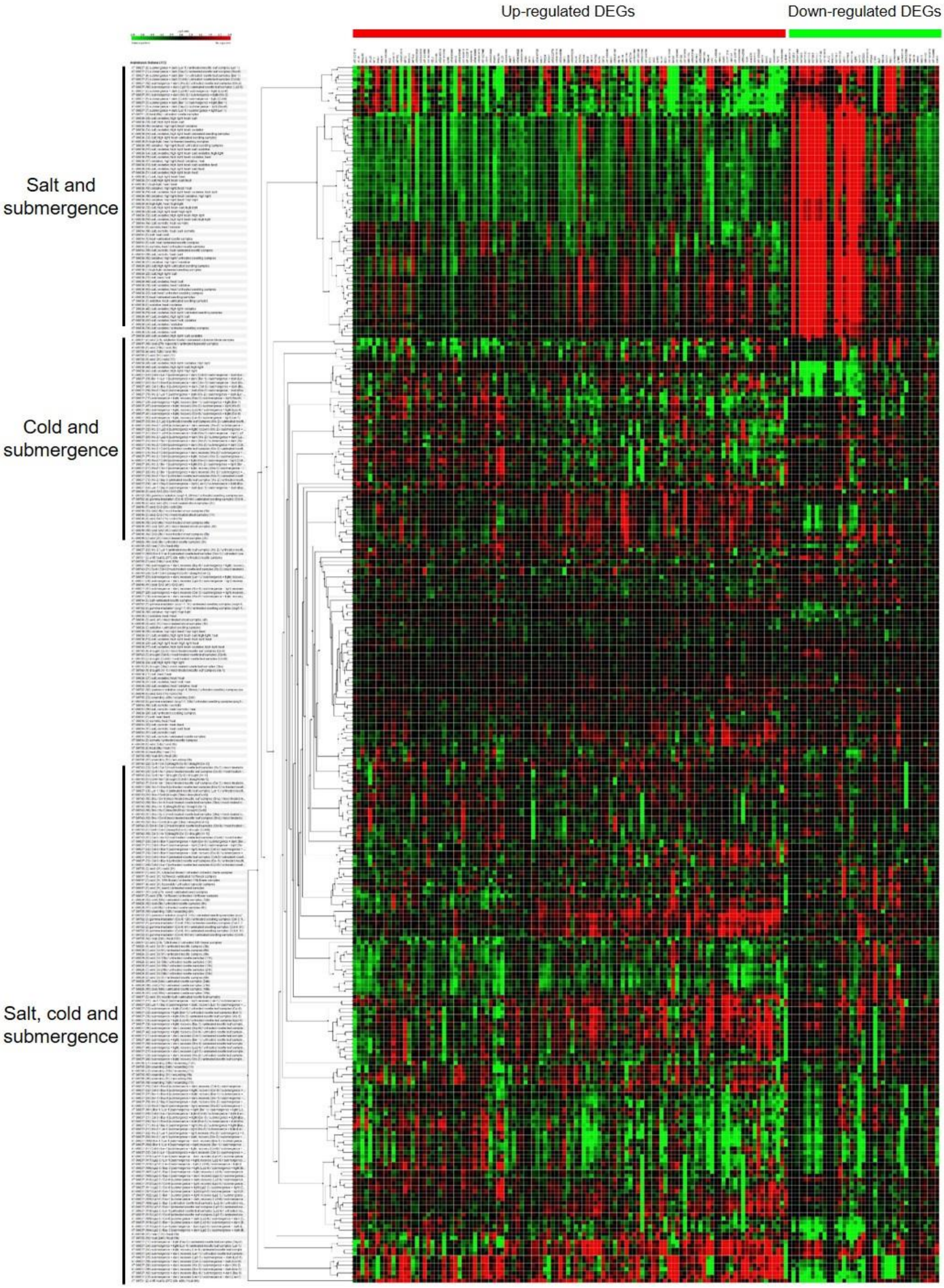

Figure S5. Hierarchical clustering analysis within Genevestigator public data. Clustering analysis was performed using the list of 151 up- (red bar) and down-regulated (green bar) DEGs (Table S1) using the clustering tool available at www.genevestigator.com. (**A**) clustering using subset “Biotic selection” with microarray studies, (**B**) clustering using subset “Biotic selection” with mRNAseq studies, (**C**) clustering using subset “Elicitor selection” with microarray studies, (**D**) clustering using subset “Elicitor selection” with mRNAseq studies, (**E**) clustering using subset “Stress selection” with microarray studies, (**F**) clustering using subset “Stress selection” with mRNAseq studies.
