# Supplementary material for: The Same against Many: AtCML8, a Ca2+ Sensor Acting as a Positive Regulator of Defense Responses against Several Plant Pathogens
Source: Int J Mol Sci. 2021 Sep 28;22(19):10469. doi: 10.3390/ijms221910469 (PMC8508799; doi:10.3390/ijms221910469)
Supplement: Supplementary file 1 [file ijms-22-10469-s001.zip › Figure S6.pdf]

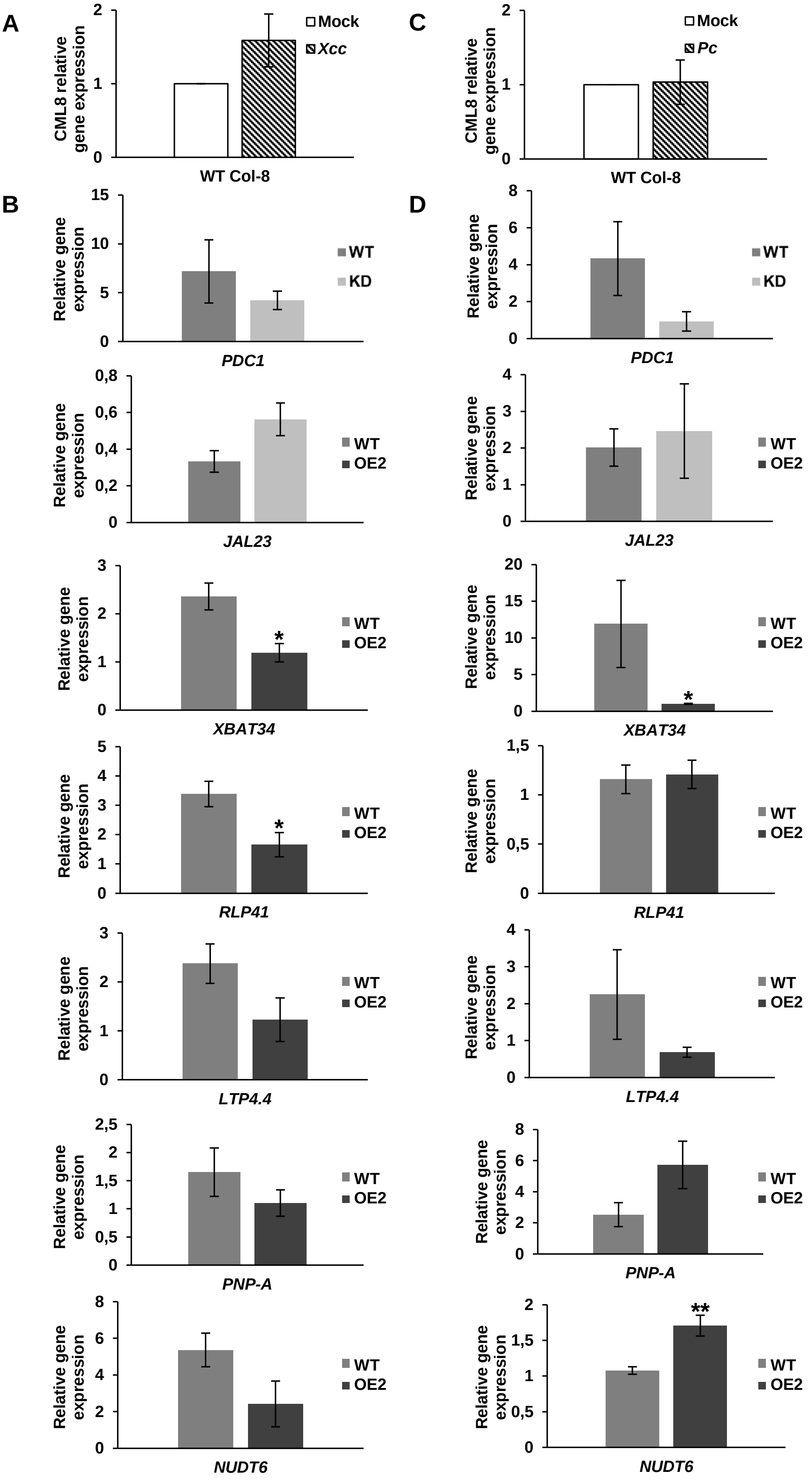

Figure S6. Expression levels of *CML8* and seven candidate genes during *Xcc* and *Pc* inoculations. RT-qPCR data show the relative gene expression of (**A**) *CML8* and (**B**) seven selected transcripts in WT (medium grey), KD (light grey) or OE2 (dark grey) at 24 hpi in *Xcc*-inoculated leaves, and (**C**) *CML8* and (**D**) seven selected transcripts in WT (medium grey), KD (light grey) or OE2 (dark grey) at 24 hpi in *Pc* inoculated roots. Transcript levels were normalized with *EF1-α* determined for the same samples. Data are presented as means of three technical replicates of two independent biological replicates. Normalized ($2^{-\Delta\Delta Ct}$) values for each differentially expressed gene were calculated as described in Materials & Methods. Mock levels thus correspond to 1. Statistical analyses were performed using Student's t-test and significant difference was found with p-values < 0.05 (*** < 0.001, ** < 0.01, * < 0.05).
