# Supplementary material for: The Same against Many: AtCML8, a Ca2+ Sensor Acting as a Positive Regulator of Defense Responses against Several Plant Pathogens
Source: Int J Mol Sci. 2021 Sep 28;22(19):10469. doi: 10.3390/ijms221910469 (PMC8508799; doi:10.3390/ijms221910469)
Supplement: Supplementary file 1 [file ijms-22-10469-s001.zip › Figure S7.pdf]

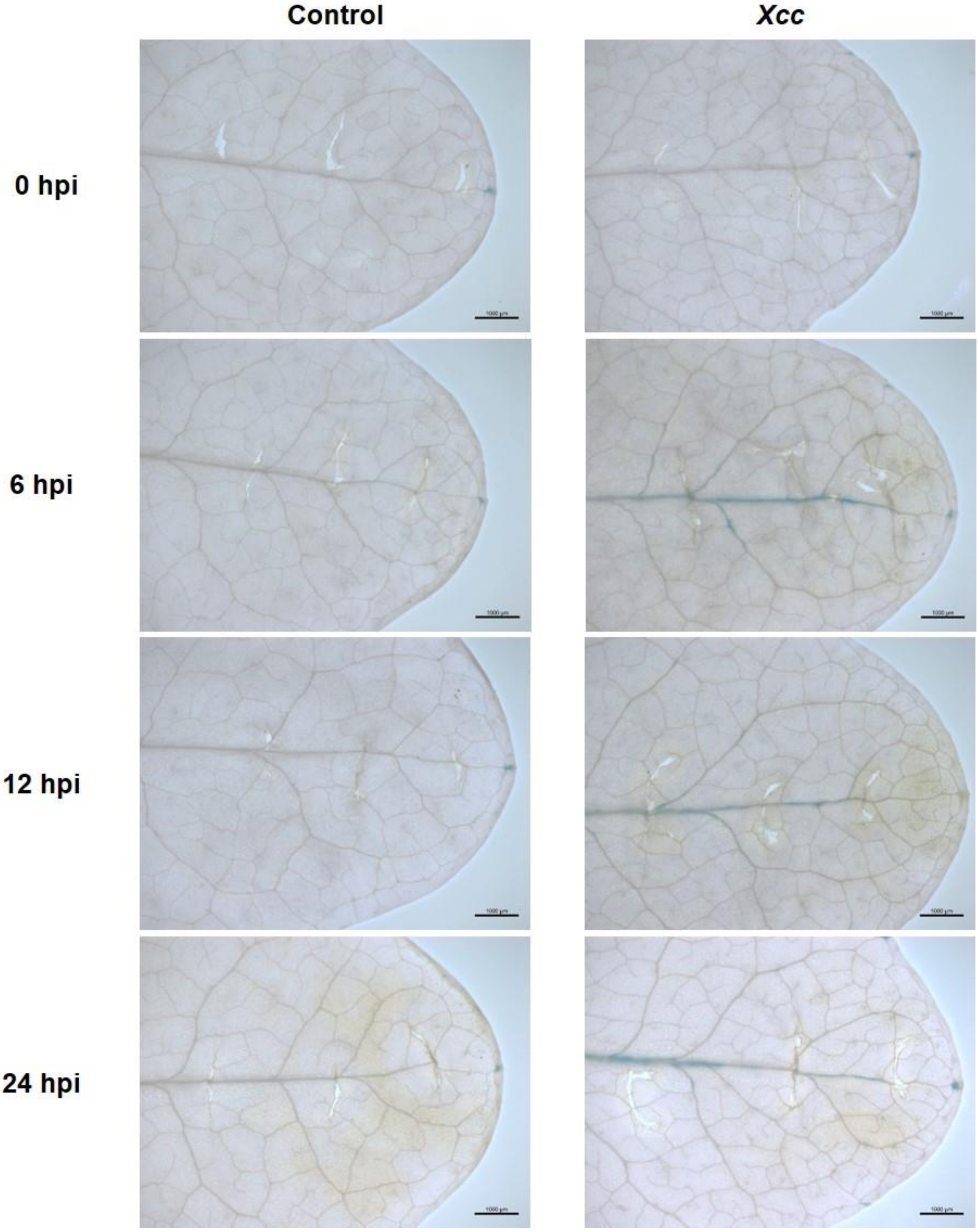

Figure S7. *CML8* gene expression in response to *Xcc* inoculation. Expression pattern of *CML8* using leaves of *promoter CML8::uidA* four weeks-old transgenic *A. thaliana* plants. GUS staining was performed 6 h, 12 h and 24 h after *Xcc* Δ*xopAC* strain (10^8 cfu.mL^{-1}) or mock treatment (Control) inoculation. Bars: 1000 µm.
