# Supplementary material for: The Same against Many: AtCML8, a Ca2+ Sensor Acting as a Positive Regulator of Defense Responses against Several Plant Pathogens
Source: Int J Mol Sci. 2021 Sep 28;22(19):10469. doi: 10.3390/ijms221910469 (PMC8508799; doi:10.3390/ijms221910469)
Supplement: Supplementary file 1 [file ijms-22-10469-s001.zip › Figure S8.pdf]

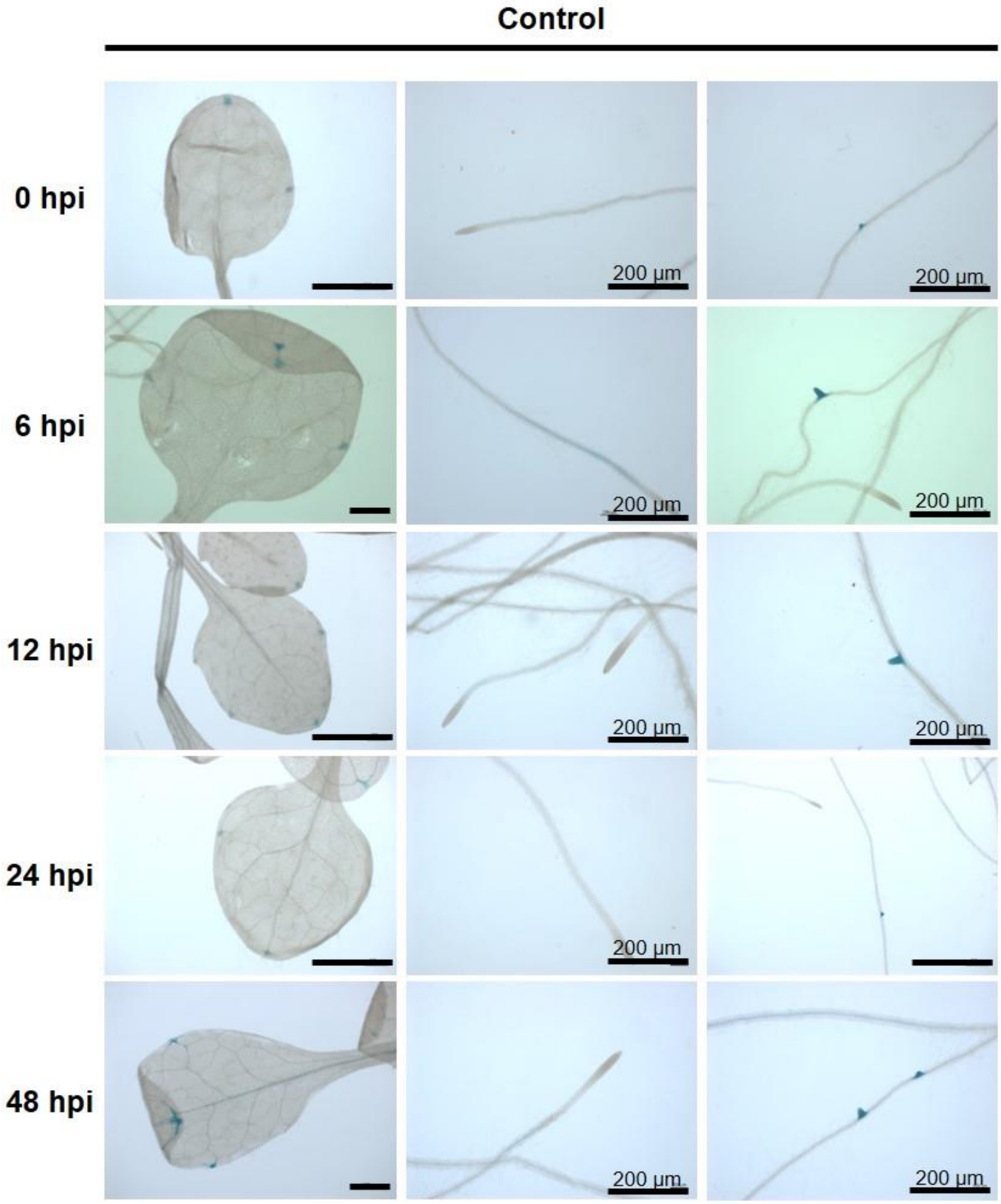
Control
0 hpi
6 hpi
12 hpi
24 hpi
48 hpi
200 µm

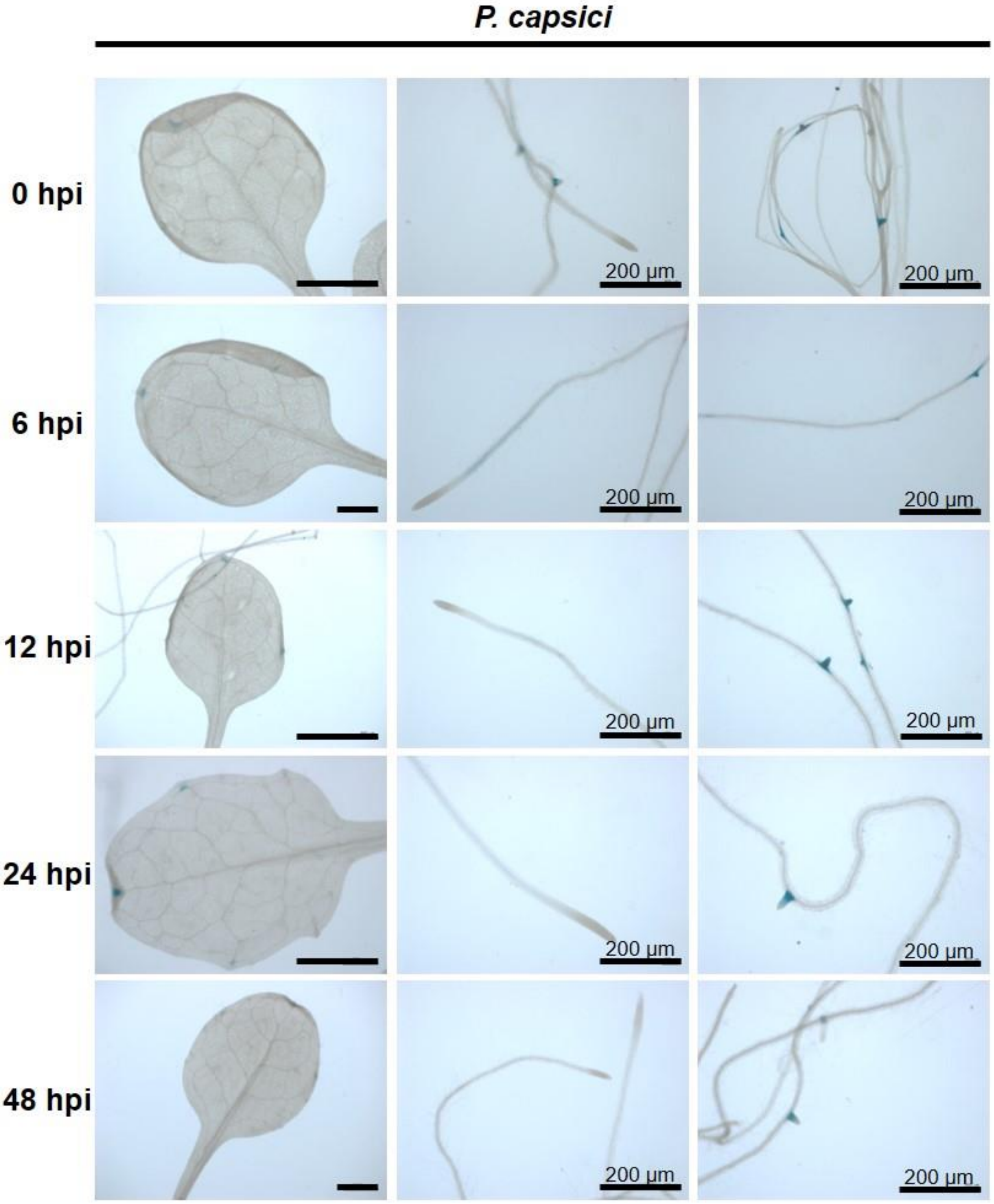

Figure S8. *CML8* gene expression in response to *Pc* inoculation. Expression pattern of *CML8* using *promoter CML8::uidA* two weeks-old transgenic *A. thaliana* seedlings. GUS staining was performed 6 h, 12 h, 24 h and 48 h after 10^3 spores.mL^{-1} *Pc* strain LT3112 or mock treatment (Control) inoculation. Bars without inscription: 500 µm.
